# Sex Pheromone Evolution Is Associated with Differential Regulation of the Same Desaturase Gene in Two Genera of Leafroller Moths

**DOI:** 10.1371/journal.pgen.1002489

**Published:** 2012-01-26

**Authors:** Jérôme Albre, Marjorie A. Liénard, Tamara M. Sirey, Silvia Schmidt, Leah K. Tooman, Colm Carraher, David R. Greenwood, Christer Löfstedt, Richard D. Newcomb

**Affiliations:** 1 The New Zealand Institute for Plant and Food Research Limited, Auckland, New Zealand; 2 Allan Wilson Centre for Molecular Ecology and Evolution, Palmerston North, New Zealand; 3 Department of Biology, Lund University, Lund, Sweden; 4 School of Biological Sciences, University of Auckland, Auckland, New Zealand; University of Michigan, United States of America

## Abstract

Chemical signals are prevalent in sexual communication systems. Mate recognition has been extensively studied within the Lepidoptera, where the production and recognition of species-specific sex pheromone signals are typically the defining character. While the specific blend of compounds that makes up the sex pheromones of many species has been characterized, the molecular mechanisms underpinning the evolution of pheromone-based mate recognition systems remain largely unknown. We have focused on two sets of sibling species within the leafroller moth genera *Ctenopseustis* and *Planotortrix* that have rapidly evolved the use of distinct sex pheromone blends. The compounds within these blends differ almost exclusively in the relative position of double bonds that are introduced by desaturase enzymes. Of the six desaturase orthologs isolated from all four species, functional analyses in yeast and gene expression in pheromone glands implicate three in pheromone biosynthesis, two Δ9-desaturases, and a Δ10-desaturase, while the remaining three desaturases include a Δ6-desaturase, a terminal desaturase, and a non-functional desaturase. Comparative quantitative real-time PCR reveals that the Δ10-desaturase is differentially expressed in the pheromone glands of the two sets of sibling species, consistent with differences in the pheromone blend in both species pairs. In the pheromone glands of species that utilize (*Z*)-8-tetradecenyl acetate as sex pheromone component (*Ctenopseustis obliquana* and *Planotortrix octo*), the expression levels of the Δ10-desaturase are significantly higher than in the pheromone glands of their respective sibling species (*C. herana* and *P. excessana*). Our results demonstrate that interspecific sex pheromone differences are associated with differential regulation of the same desaturase gene in two genera of moths. We suggest that differential gene regulation among members of a multigene family may be an important mechanism of molecular innovation in sex pheromone evolution and speciation.

## Author Summary

Chemical signals are prevalent in sexual communication systems, especially within the Lepidoptera where sex pheromones are typically one of the defining characteristics of species. We have isolated six desaturases from two groups of sibling species of leafroller moths belonging to the genera *Ctenopseustis* and *Planotortrix*. Functional analyses in yeast and quantitative RT–PCR indicate that three of the desaturases are involved in the biosynthesis of sex pheromone components in these species. One of three enzymes is a Δ10-desaturase that is differentially expressed in the pheromone glands of the two sets of sibling species, consistent with differences in the pheromone blend in both species pairs. In the pheromone glands of species that utilize (*Z*)-8-tetradecenyl acetate as sex pheromone component (*C. obliquana* and *P. octo*), the expression levels of the Δ10-desaturase are significantly higher than pheromone gland expression levels in their sibling species (*C. herana* and *P. excessana*). Our results demonstrate that interspecific sex pheromone differences are associated with differential regulation of the same desaturase gene in these two genera of moths. Based on these findings differential gene regulation among members of a multigene family may be an important mechanism of molecular innovation in sex pheromone evolution and speciation.

## Introduction

Variation is the raw material of evolution; however the nature of this variation remains a topic of much discussion amongst evolutionary biologists [1], [2], [3]. The potential role in evolution of mutations that impact gene regulation rather than the amino acid sequence of a protein was initially proposed in the 1970s. King & Wilson [4] noticed that orthologous proteins between humans and chimpanzees were very similar at the amino acid level compared to the phenotypic differences between the two species and suggested that gene regulation could play an important role in explaining some of the phenotypic differences between the two primates. Since then the relative importance in evolution of regulatory mutations (those affecting gene expression) versus structural mutations (those resulting in amino acid substitutions within the coding region of a protein) has been debated (e.g., Hoekstra and Coyne [1] and references therein).

While structural mutations between orthologous proteins are easy to identify, mutations that affect the regulation of a gene are more difficult to recognize. Regulatory mutations can fall close to the gene in its promoter region (*cis*-regulatory mutations) or act at a distance from the gene (*trans*-regulatory mutations). *Cis*-regulatory mutations usually result in the gain or loss of a site involved in binding a regulatory factor, such as a transcription factor, whereas *trans*-acting regulatory mutations typically affect the transcription factors themselves. Interestingly, *trans*-acting regulatory mutations can involve both regulatory and structural mutations of the transcription factor. The relative importance of *cis*- versus *trans*-regulatory mutations in the course of evolution is also predicted to be influenced by the degree of pleiotropy [5]. Since transcription factors generally influence the expression of multiple genes, *trans*-regulatory mutations are more likely to affect a number of traits. On the other hand, *cis*-regulatory mutations are more likely to impact the target gene alone [6]. In spite of this, both types of regulatory mutations have been identified in the evolution of distinct cases of beneficial traits within species [1] and in the evolution of major morphological innovations at higher taxonomic levels [7]. Despite these examples, there remains little evidence regarding the nature of molecular innovations that underpin the evolution of new species. Few genes involved in speciation have been identified to date [8], and many causal mutations associated with species differences remain unknown. This makes the topic of great interest to further investigate the role of structural mutations and gene regulation in traits that can lead to speciation. Finally, whether these mutations are present as standing variation in the ancestral species or if the process of speciation is limited by the rate of acquisition of newly arising mutations has only recently attracted attention, with most discussion restricted to the acquisition of beneficial traits within species [9], [10].

Speciation is often associated with changes in mate recognition systems [11]. Mate recognition has been extensively studied within the Lepidoptera where the production of long-range species-specific sex pheromone signals by females and their recognition by conspecific males are critical steps. Furthermore, for many species within the Lepidoptera, sex pheromones are often the defining character for biological species [12]. The sex pheromones of many species of moths have been identified [13], and in some systems, enzymes involved in the biosynthesis of pheromone components have been characterized [14], [15]. An important structural characteristic of moth sex pheromone components is the position of double bond(s) along the fatty acid backbone of the molecule. These double bonds are introduced at specific carbon positions by distinct members of the fatty-acyl desaturase family that have evolved a role in pheromone biosynthesis from an ancestral function in essential fatty-acid biosynthesis [16]. A core set of desaturase transcripts is typically found in the pheromone glands of female moths of a given species [16], including two Δ9-desaturases, one with a preference for 16 carbon (16C) fatty acids over 18 carbon (18C) fatty acids and a second with the opposite preference, together with one or several members of the so-called Δ11-desaturase clade that includes enzymes that are increasingly being shown to possess a wide range of desaturation abilities (e.g. Liénard et al. [17] and references therein).

Changes in enzymes involved in pheromone biosynthesis, including desaturases, have been implicated in pheromone differences in both moths and flies. In the Lepidoptera, these include examples of gene neofunctionalisation, where new desaturases have arisen by gene duplication and then diverged to evolve a new function [18], [19]. In contrast, there is a single example where differential expression of desaturase genes in the Asian corn borer is responsible for producing distinct pheromone blends [20]. In *Drosophila* differential regulation of desaturase F is implicated in the production of distinct cuticular hydrocarbon pheromones between species [21]. With only a few cases to draw from, the importance of structural versus regulatory changes involved in mate recognition and speciation is an open question and more examples are required to form a consensus of their relative contribution.

To investigate the role of desaturases in changes in sex pheromone blends, we have studied the mode of evolution in these enzymes within two genera of leafroller moths, *Ctenopseustis* (brown-headed leafrollers) and *Planotortrix* (green-headed leafrollers). Both genera are endemic to New Zealand, with species within the two genera widely distributed across the two main islands [22]. Sequence divergence at the COI locus averages 10%, suggesting that the two genera diverged around 5 million years ago [23]. Although some species develop on specific host plants, such as *P. aviciennae* on mangroves (*Avicienna marina*) or *C. fraterna* on silver fern (*Cyathea dealbata*), most of the species within the genera are polyphagous, and can develop on angiosperms or gymnosperms, including a number of horticultural and silvicultural crops. Many of the sibling species are difficult to differentiate using classical morphology [22] or mitochondrial bar-coding DNA markers [23], suggesting that they have diverged in the last 500,000 years. Despite this recent divergence, the different sibling species use distinct sex pheromones. Their sex pheromone compounds are tetradecenyl acetates that differ primarily in the position of a single double bond within a fourteen carbon fatty acid backbone. They are biosynthesized from fatty acids (myristic, palmitic or stearic acid), which are desaturated at specific positions, chain-shortened via β-oxidation, reduced to fatty alcohols, and acetylated to give the final products. Within the genera sex pheromone blends contain up to three components in specific ratios (Table S1) that are desaturated at the Δ5, Δ7, Δ8, Δ9 or Δ10 positions, all in the *Z* configuration [24]. Each genus contains a pair of sibling species that utilize distinct sex pheromone blends. In *Planotortrix*, *P. excessana* uses a blend of (*Z*)-5-tetradecenyl acetate (Z5-14:OAc) and (*Z*)-7-tetradecenyl acetate (Z7-14:OAc) [25], [26], while its sibling species *P. octo*, utilizes (*Z*)-8-tetradecenyl acetate (Z8-14:OAc) and trace amounts (2%) of (*Z*)-10-tetradecenyl acetate (Z10-14:OAc) [25]. In *Ctenopseustis*, *C. obliquana* uses a blend of Z5-14:OAc and Z8-14:OAc [27], while its sibling species *C. herana* utilizes a sex pheromone consisting solely of Z5-14:OAc [28]. Thus each species pair is characterized by a gain or loss of a particular pheromone component that differs only in the position of a double bond.

Sex pheromones are central to mate recognition in moths and form barriers to gene flow among the Lepidoptera. Species within *Ctenopseustis* and *Planotortrix* are no exception. Males of these species are specifically attracted to the sex pheromone blend of their conspecific females, but not to that produced by the respective sibling species, restricting gene flow between sibling species (see Foster et al. [24] for a review). Briefly, Clearwater et al. [29] investigated the cross responses of *C. obliquana* and *C. herana* males to conspecific and sibling species sex pheromone blends, as well as females, in wind tunnel and field cage experiments. They found strong preferences for the conspecific pheromone in both experimental formats. Furthermore, Foster et al. [30] tested the attractiveness of a range of ratios of Z5-14:OAc and Z8-14:OAc to *C. obliquana* and *C. herana* in a wind tunnel. *C. obliquana* never landed on lures containing only Z5-14:OAc, while *C. herana* males never landed on lures containing Z5-14:OAc and Z8-14:OAc. Similarly, field cage cross attraction experiments using *P. excessana* and *P. octo* showed high species specificity in male mating behaviour [25]. The lack of interbreeding in the wild is also supported by isozyme-based population genetics, with at least one fixed difference identified for each sibling species pair [31], [32].

**Figure 1:**
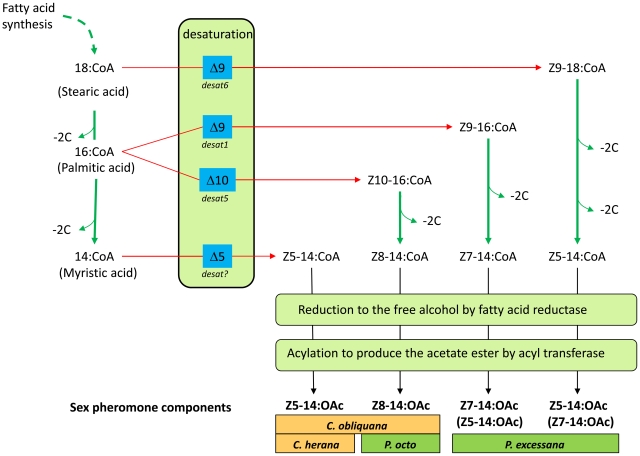
Schematic outlining the likely biosynthetic routes of the sex pheromone components of *C. obliquana*, *C. herana*, *P. octo*, and *P. excessana*. *Desat1*, *desat5* and *desat6* correspond to the desaturase genes encoding a Δ9 desaturase with a preference for 16>18 carbon fatty acids, a Δ10-desaturase and a Δ9-desaturase with a preference for 18>16 carbon fatty acids, respectively. *Desat?* refers to a yet to be identified Δ5-desaturase. Chain shortening by β-oxidation is indicated by ‘−2C’. The minor products of the two Δ9-desaturases in *P. excessana* (*desat1* and *desat6*) are indicated in brackets. We also note that Z10-14:OAc is a very minor (2%) component of the pheromone blend of *P. octo* (not shown).

The biosynthesis of the compounds found in the pheromone blends of the *Ctenopseustis* and *Planotortrix* species have been studied by Fatty Acid Methyl Ester (FAME) analysis of pheromone glands [33] and by monitoring the incorporation of labelled precursors [34], [35], [36]. The Z8-14:OAc used by *P. octo* is the product of Δ10-desaturation of palmitic acid followed by chain shortening, reduction and acetylation [35], while the Z5-14:OAc and Z7-14:OAc used by *P. excessana* are the products of Δ9-desaturase activity [36]. The Δ10- and Δ9-desaturases responsible for these activities have been isolated and characterized from *P. octo* [37]. The biosynthesis of Z5-14:OAc used by *Ctenopseustis* species was investigated by labelling experiments in *C. herana*, where unlike in *Planotortrix*, FAME analysis implicated the action of a specific Δ5-desaturase [34]. Therefore at least four desaturases are thought to potentially contribute to pheromone biosynthesis in these two genera: two Δ9-desaturases, a Δ10-desaturase and a Δ5-desaturase (Figure 1).

We set out to investigate whether structural mutations within coding regions of these desaturases or differential regulation of a standing set of desaturase genes are responsible for the diversity in sex pheromone components used by species within the genera *Ctenopseustis* and *Planotortrix*. To obtain a general picture of the set of genes present in these leafroller moths, we first isolated and characterized desaturases from genomic DNA and cDNA from the pheromone glands of *C. obliquana*, *C. herana*, *P. octo* and *P. excessana*. We then performed functional assays and quantitative real-time PCR to identify the desaturases likely to have a role in sex pheromone biosynthesis in these species. Finally, we compared coding region sequences and the expression of the genes in the pheromone glands of the sibling species to address our question.

## Results

### Isolation and phylogenetic analysis of desaturase genes

Initially we isolated as many desaturase-encoding sequences as possible from two sets of sibling species in the genera *Ctenopseustis* and *Planotortrix*. By Polymerase Chain Reaction (PCR) using degenerate primers designed to target conserved regions of lepidopteran fatty acyl desaturases, we isolated 29 desaturase-like sequences from genomic DNA and/or pheromone gland cDNA of *C. obliquana*, *C. herana*, *P. excessana*, *P. octo* and in some instances, also from the more distantly related *Planotortrix* species, *P. notophaea*. For many of these genes, sequences were recovered from two strains of *P. excessana* (the North Island and South Island strains). The sequences fall into six groups of desaturase-like genes (*desat1-6*). Rapid Amplification of cDNA Ends (RACE) PCR was used together with genome walking and analysis of preliminary whole genome sequencing assemblies of *C. obliquana* and *P. octo* (unpublished data) to construct predicted coding regions for each ortholog of each desaturase-like gene. The resulting gene contigs were confirmed by PCR and sequencing from pheromone gland cDNA wherever possible. Coding regions were obtained for all genes from all species except for *desat3* and *desat5* from *C. herana*, and the final 3′ ∼100 bp of *desat5* from *C. obliquana*. *Desat1* and *desat5* of *P. octo* were isolated previously by Hao et al. [37]. All sequences isolated during this study have been deposited on GenBank (accession numbers JN022472–JN022498). An amino acid alignment derived from the 29 sequences grouped into each of the six desaturase-like genes is presented in Figure S1.

The predicted desaturases for which we obtained full length coding regions ranged in size from 331 to 358 amino acids (Table 1, Text S1, S2, S3, S4, S5, S6). Between species (excluding *P. notophaea*), amino acid sequence identities were highest for *desat6* (98.6%–100%) followed by *desat1* (97.4%–99.4%) and *desat2* (96.1%–99.4%), with the lowest displayed by *desat3* (90.4%–95.5%) and *desat4* (90.2%–96.8%). The full length sequence of the *desat5* was only obtained for *Planotortrix* species, where the amino acid identity between sibling species was 99.4%. Intron positions were inferred by PCR from genomic DNA or in the case of *C. obliquana* and *P. octo* by direct observation from genome scaffolds. Where determined, intron positions were conserved among orthologous genes. *Desat2* contains no introns, *desat4* contains two introns and the remaining desaturase-like genes, *desat1*, *desat3*, *desat5* and *desat6* each contain three introns. The relative positions and phase of the introns are indicated on the alignments presented in Text S1, S2, S3, S4, S5, S6.

**Table 1:** Summary statistics for desaturases from *Ctenopseustis* and *Planotortrix* species.

	s^a^	N^b^	S^c^	k^d^	ωM0^e^	ωM3^f^
*desat1*	5	352	0.332	1.87	0.035	0.035
*desat2*	4	331	0.313	2.37	0.056	0.056
*desat3*	3	332	0.566	1.42	0.111	0.124
*desat4*	3	349	0.296	4.54	0.233	0.233
*desat5*	6	358	0.839	2.33	0.391	0.472
*desat6*	6	353	0.421	3.08	0.526	0.135

a number of sequences.

b number of codons.

c tree length.

d transition/transversion ratio.

e ω under M0.

f ω under M3.

**Figure 2:**
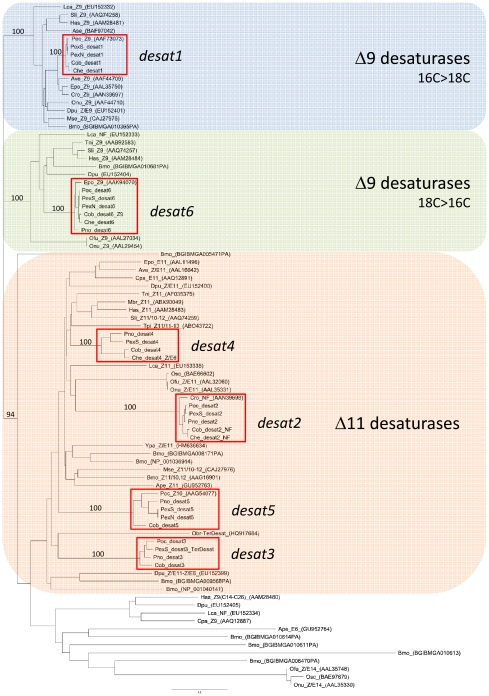
Phylogeny of 86 lepidopteran desaturases including those encoded by *desat1-6* from *Ctenopseustis* and *Planotortrix*. The phylogeny was constructed from protein sequences using PHYML implemented within Geneious using JTT distances. Complete amino acid sequence information was obtained from GenBank, along with desaturases predicted from the genomic sequence of *Bombyx mori* from the Silkmoth database. Sequences are abbreviated as following: Ape, *Antherea pernyi*; Ase, *Ascotis selenaria*; Ave, *Argyrotaenia velutinana*; Bmo, *Bombyx mori*; Cpa, *Choristoneura parallela*; Cro, *Choristoneura rosaceana*; Che, *Ctenopseustis herana*; Cob, *Ctenopseustis obliquana*; Cpo, *Cydia pomonella*; Epo, *Epiphyas postvittana*; Has, *Helicoverpa assulta*; Hze, *Helicoverpa zea*; Lca, *Lampronia capitella*; Mbr, *Mamestra brassicae*; Mse, *Manduca sexta*; Pex, *Planotortrix excessana*; Poc, *Planotortrix octo*; Pno, *Planotortrix notophaea*; Onu, *Ostrinia nubilalis*; Ofu, *Ostrinia furnacalis*; Osc, *Ostrinia scapulalis*: Obr, *Operophtera brumata*; Sli, *Spodoptera littoralis*; Tni, *Trichoplusia ni*; Tpi, *Thaumetopoea pityocampa*; Ypa: *Yponomeuta padellus*. After the abbreviated species name are the desaturase activity if known with NF  =  non-functional in pheromone biosynthesis; TerDesat  =  terminal desaturase activity; Z or E, geometry of the double bond. The GenBank accession numbers are given in brackets for previously described desaturases. Bootstrap values in percentages from 1000 bootstrap replicates supporting the three major clades (Δ9-desaturase 16C>18C, Δ9-desaturase 18C>16C, and Δ11-desaturase) and the groups containing the *Ctenopseustis* and *Planotortrix* desaturases, indicated by the red outline boxes, are given above the relevant branches.

Phylogenetic analysis was conducted on all predicted desaturases from *Ctenopseustis* and *Planotortrix* reported above or previously [37], as well as a set of currently available lepidopteran desaturase sequences (Figure 2). Three well-supported clades were observed including a Δ9-desaturase (16C>18C) clade into which the predicted protein desat1 falls, a Δ9-desaturase (18C>16C) clade into which desat6 falls and a so-called Δ11-desaturase clade into which all the remaining *Ctenopseustis* and *Planotortrix* desaturases (desat2, desat3, desat4 and desat5) fall. In each case, the *Ctenopseustis* and *Planotortrix* orthologs group together, well supported by high bootstrap values. Moreover, some *Ctenopseustis* and *Planotortrix* desaturases group closely with previously characterized desaturases from other species. First, the *Ctenopseustis* and *Planotortrix* desat2 orthologs group closely with a non-functional desaturase from *Choristoneura rosaceana* [38]; second, the desat6 orthologs group with a Δ9-desaturase from *Epiphyas postvittana* [39]; and third, even though less closely related, desat3 groups with a terminal desaturase from *Operophtera brumata* [40]. Finally, the six predicted desaturases from *Ctenopseustis* and *Planotortrix* are all well separated from each other in the phylogenetic tree, with many other lepidopteran desaturases inter-dispersed between them.

We looked for evidence of nonsense or missense mutations in the sequence of the desaturase-like genes that might impact function and explain differences in the pheromone components used by the different species. No amino acid substitutions were found in the active site regions, such as the histidine-rich regions involved in ion coordination, and no premature stops or frame-shift mutations could be identified. We then undertook likelihood ratio tests using PAML to look for evidence of selection acting on the coding regions of the six desaturases [41]. There was some evidence for positive selection in one of three model comparisons for *desat3* and *desat5* (Table 2), together with some significantly selected sites identified in *desat3* (7), *desat4* (2), *desat5* (1) and *desat6* (1) (Table 3). However, the ratio of non-synonymous to synonymous (dN/dS) nucleotide substitutions (M0) were less than one for all genes, indicative of overall purifying selection and suggestive of conserved function (Table 1).

**Table 2:** Likelihood ratio tests between nested site-specific models.^a^

	2Δ*l* a		2Δ*l*		2Δ*l*	
gene	M0 v. M3	sig	M7 v. M8	sig	M8a v. M8	sig*^b^*
*desat1*	0	NS	0.217	NS	0.121	NS
*desat2*	0	NS	0.001	NS	0	NS
*desat3*	10.706	*p* = 0.03	3.303	NS	0	NS
*desat4*	0	NS	0	NS	0	NS
*desat5*	3.356	NS	3.877	NS	4.151	*p* = 0.04
*desat6*	5.513	NS	3.877	NS	0	NS

a Twice the difference of log likelihood between the two models (χ^2^)  =  probability that two models should differ in log likelihood, as much as that observed, given the degree of freedom. Degrees of freedom are equal to the difference in the number of parameters between models; M0 v. M3  =  4, M7 v. M8  = 2, M8a v. M8  = 2, M8a v. M8  = 1. M8a v. M8 comparison significance is determined by a *P*-value for the 50∶50 mixture of distributions.

**Table 3:** Putative positively selected sites and posterior probabilities under M8.

gene	Site #	M8ω	M8 (BEB) posterior probability	Amino acids at selected site
*desat1*	-	-	-	-
*desat2*	-	-	-	-
*desat3*	14		0.593	E,A,D
	43		0.730	L,I
	54		0.713	S
	101		0.611	F,M
	305		0.741	V,L,A
	316		0.714	G,L
	320		0.755	E,S
*desat4*	12		0.610	K,E,D
	162		0.618	K,T
*desat5*	11		0.503	Q,R,C
*desat6*	97		0.817	S

### Functional analysis of desaturases in yeast

We then examined the function of the predicted desaturases, or more specifically substrate preference and desaturation specificity within the fatty acid precursors. We investigated sufficient desaturases so that at least one ortholog of each of the six predicted desaturases was characterized. The open reading frames of predicted desaturases were subcloned into the YEpOLEX or pYEX-expression vectors and functional expression was conducted in desaturase-deficient yeast strains. FAME extracts from transformed yeast were analysed to infer the ability of each desaturase to introduce double bonds to pheromone precursors at specific positions and DiMethyl-DiSulphide (DMDS) derivatization was performed to verify the structural identity of the unsaturated products. Since functional analyses from *P. octo* have shown that desat1 has Δ9-desaturase activity with a preference for 16C over 18C precursors, and desat5 is a Δ10-desaturase [37], we focused on orthologs of desat2, desat3, desat4 and desat6.

**Figure 3:**
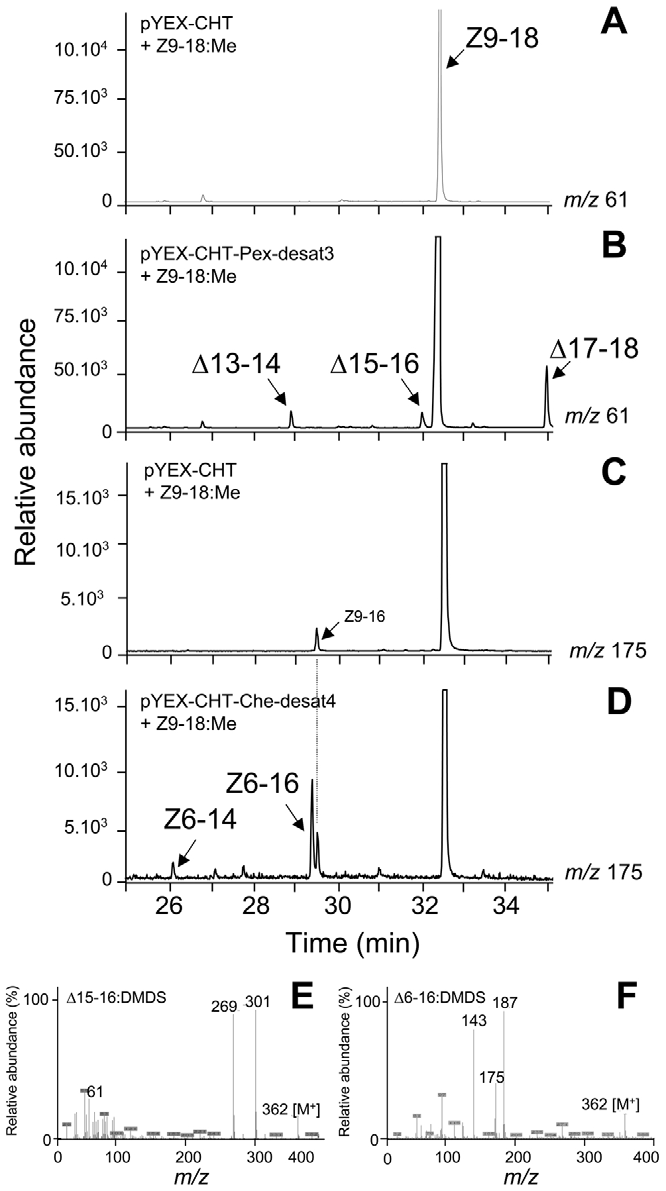
GC-MS analyses. GC-MS analyses of DMDS derivatives from methanolysed Cu^2+^-induced *ole1 elo1 S. cerevisiae* yeast supplemented with Z9-18:Me and transformed with (A and C) control pYEX-CHT vector, (B) pYEX-CHT-Pex-desat3 and (D) pYEX-CHT-Che-desat4. The chromatogram traces represent the ion currents obtained by selection of the characteristic ion of terminal and Δ6-DMDS adducts at *m/z* 61 (A and B) and *m/z* 175 (C and D), respectively. (E) and (F) represent the mass spectra for terminal C16 DMDS adducts (Δ15-16) (*m/z* 362 [M^+^], 61, 301 (A^+^) and 269 (A^+^-32)) and Δ6-16 DMDS adducts (*m/z* 362 [M^+^], 187, 175 (A+) and 143 (A^+^-32), respectively. The mass spectra for other DMDS adducts present in the extracts are not shown and displayed a spectrum with the characteristic ions at *m/z* 334 [M^+^], 61 and 273 for Δ13-14, at *m/z* 390 [M^+^], 61 and 329 for Δ17-18 and at *m/z* 334 [M^+^], 175 and 159 for Δ6-14, respectively.

Desat2 from *C. obliquana* or *C. herana* had the same profile as Cu^2+^-induced yeast transformed with the pYEX-CHT-only vector (Figure 3A), indicating that they were unable to utilize typical sex pheromone precursors as substrates.

FAME analyses from yeast transformed with *desat3* from *P. excessana* and *desat4* from *C. herana* revealed the presence of three and two additional mono-unsaturated products, respectively. In the case of *desat3*, all DMDS adducts exhibited a diagnostic ion at *m/z* 61, corresponding to FAMEs with a terminal double bond. These were subsequently identified as the Δ13-14:Me (*m/z* 334 [M^+^], 61 and 273), the Δ15-16:Me (*m/z* 362 [M^+^], 61 and 301) (Figure 3B–3E) and the Δ17-18:Me (*m/z* 390 [M^+^], 61 and 329) (Figure 3B). The DMDS adducts for *desat4* exhibited a diagnostic ion at *m/z* 175, which is characteristic of FAMEs with a double bond at the sixth carbon position (Δ6) and was absent in control samples (Figure 3C). The DMDS adducts corresponded to the Δ6-14:Me (*m/z* 334 [M^+^], 175 and 159) (Figure 3D) and Δ6-16:Me (*m/z* 362 [M^+^], 175 and 187) (Figure 3D, 3F). No other characteristic ions for mono-unsaturated compounds were detected.

**Figure 4:**
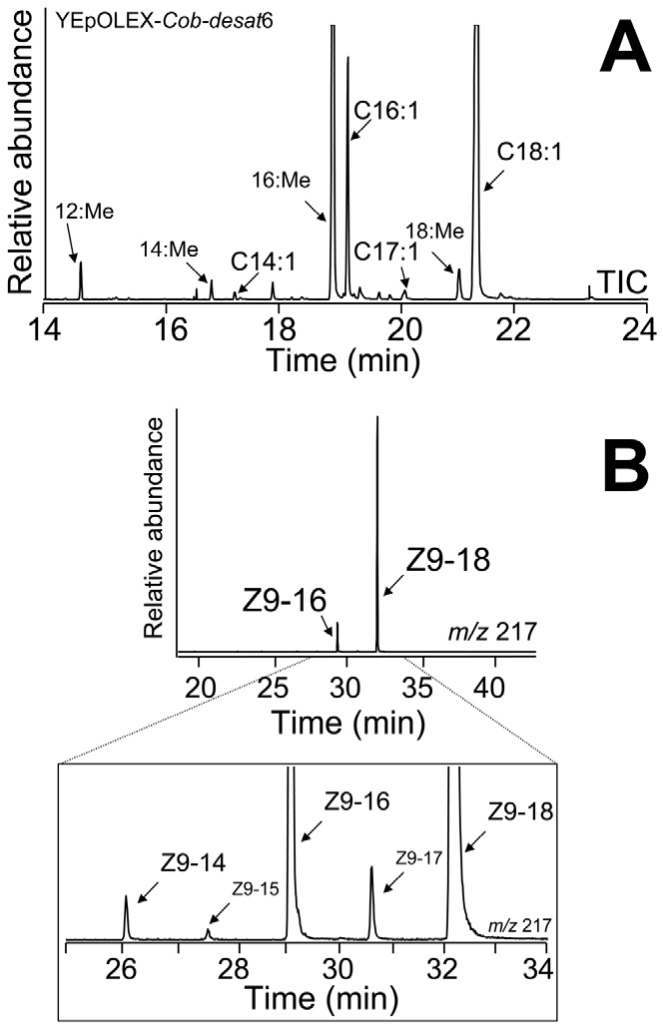
GC-MS analysis. GC-MS analysis of methanolysed total lipid extracts from *ole1 S. cerevisiae* yeast transformed with YEpOLEX-Cob-desat6. (A) Total ion current (TIC) chromatogram of fatty acid methyl esters of yeast expressing the Cob-desat6 gene. (B) DMDS derivatives of methanolyzed YEpOLEX-Cob-desat6 yeast extracts. The chromatogram traces represent the ion current obtained by selection of the characteristic ion of Δ9-adducts at *m/z* 217.

YEpOLEX-Cob-desat6 yeast transformants were able to grow on media lacking Unsaturated Fatty-Acids (UFAs), indicating that *desat6* from *C. obliquana* encodes a functional desaturase that could complement the UFA auxotrophic *ole1* strain. Methylated fatty-acid extracts from yeast bearing *desat6* transformants showed two major peaks with retention times corresponding to Z9-16:Me and Z9-18:Me (Figure 4A). DMDS analyses revealed a diagnostic ion at *m/z* 217, confirming the identity of unsaturated FAMEs with a double bond at the Δ9 position. In addition, more Z9-18:Me than Z9-16:Me was produced, with small amounts of Z9-14:Me (*m/z* 334 [M^+^], 217 and 117), Z9-15:Me (*m/z* 348 [M^+^], 217 and 131) and Z9-17:Me (*m/z* 376 [M^+^], 217 and 159) also detected (Figure 4B).

We are therefore able to summarise the activities of the six predicted desaturase groups, assuming that their function is conserved among orthologs. *Desat1* encodes a Δ9-desaturase, with a preference for 16C over 18C precursors, *desat2* encodes an enzyme for which an activity has not yet been detected, *desat3* encodes a terminal desaturase, *desat4* encodes a Δ6-desaturase, *desat5* a Δ10 desaturase and *desat6* a Δ9-desaturase with a preference for 18C over 16C precursors. Based on these functional analyses we concluded that *desat1*, *desat5* and *desat6* are likely to play a role in sex pheromone biosynthesis in these species.

### Gene expression analysis of desaturase genes

Initial gene expression analysis using quantitative real-time PCR conducted from cDNA from pooled tissue samples (approx. 100 pheromone glands) by species was undertaken on all six desaturases to examine relative gene expression in larval fat bodies, adult male and female abdomens and adult female pheromone glands. The apparently non-functional *desat2* was highly expressed only in larval fat bodies, while all other desaturases had detectable levels of expression across all tissues with *desat1*, *desat5* and *desat6*, showing high levels of expression in the pheromone gland (data not shown). Because of these results and the unlikely involvement of *desat2*, *desat3* and *desat4* in pheromone biosynthesis based on their desaturase activities, we focused on the analysis of the expression levels of *desat1*, *desat5* and *desat6*, comparing gene expression from pheromone glands of individual female moths. Our hypothesis was that expression differences among the desaturases are responsible for the observed pattern of sex pheromone component differences between the sibling species.

**Figure 5:**
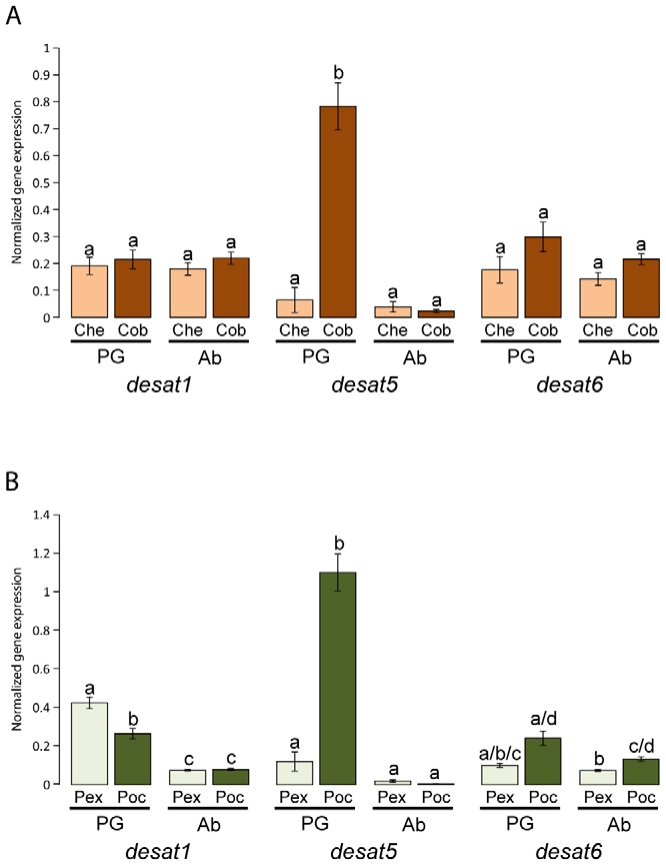
Gene expression of *desat1*, *desat5*, and *desat6* in the pheromone gland and abdomen of virgin females in *Ctenopseustis obliquana*, *C. herana*, *P. excessana*, and *P. octo* relative to housekeeper genes. In panel (A) the normalised expression levels in the pheromone gland [PG] of *C. herana* (Che; light brown; n = 20) and *C. obliquana* (Cob; dark brown; n = 21) was compared with those in the abdomen [Ab] of *C. herana* (n = 20) and *C. obliquana* (n = 21), while in panel (B) the normalised expression levels in the pheromone gland of *P. excessana* (Pex; light green; n = 39) and *P. octo* (Poc; dark green; n = 24) are compared with those in the abdomen of *P. excessana* (n = 39) and *P. octo* (n = 25). Bars are the mean normalized gene expression, with error bars representing SEMs. Different small case letters indicate significant differences between tissues and/or species at the 95% level using the Bonferroni correction for each desaturase gene.

The most striking expression differences in the pheromone glands compared with adult abdomens of the different species among the three desaturases were observed for *desat5* (Figure 5). Levels of gene expression were higher in *C. obliquana* (34-fold) and *P. octo* (273-fold) pheromone glands compared with abdomens than in *C. herana* and *P. excessana*, respectively, where no significant differences in expression were observed between both tissues. Differences in expression of *desat1* and *desat6* between species and tissues were also observed, but were far less striking than for *desat5*. *Desat1* was more highly expressed in the pheromone glands than in abdomens of *P. excessana* (3.7-fold) and *P. octo* (3.3-fold), while for *desat6 P. octo* showed higher levels of expression than *P. excessana* both in the gland and the abdomen (1.8-fold). No significant differences in *desat1* and *desat6* expression were found in the two *Ctenopseustis* species. To verify that the primers used for quantitative RT-PCR were able to amplify the appropriate desaturase gene with low or barely detectable levels of expression in a particular species (for example *desat5* from *C. herana* and *P. excessana*), PCRs were conducted using genomic DNA or plasmids containing the relevant desaturase cDNA. In all cases, melting-curve analysis confirmed the presence of a single product of the expected size and/or sequence and negative controls contained no product (data not shown).

## Discussion

The molecular mechanisms involved in the production of the variants that underpin the evolution of new mate recognition systems and new species remain poorly understood. Toward addressing this, we have investigated the molecular differences in desaturase genes involved in pheromone biosynthesis in sibling species within the *Ctenopseustis* and *Planotortrix* genera of leafroller moths. We were particularly interested in whether differential regulation of a standing set of desaturase genes within a multigene family is involved in the observed differences in sex pheromone composition between the sibling species, *C. obliquana* vs. *C herana* and *P. octo* vs *P. excessana*.

Initially, we set out to identify the sequences encoding the four desaturases previously identified biochemically within the pheromone glands of these species. In total we isolated 27 new lepidopteran desaturase-like sequences from five species within the leafroller moth genera *Ctenopseustis* and *Planotortrix* (*C. obliquana*, *C. herana*, *P. octo*, *P. excessana* and *P. notophaea*). Together with the two desaturases previously isolated and characterized from *P. octo* [37], these 29 sequences represent six orthologous desaturases. We examined the ability of a representative subset of these predicted desaturases to introduce double bonds in sex pheromone precursors such that at least one member of each set of six orthologous desaturases has now been functionally characterized.

Two of the six desaturases from *Ctenopseustis* and *Planotortrix* encode Δ9-desaturases (*desat1* and *desat6*) that display highly conserved functions across all lepidopteran insects investigated to date [17], [19], [37], [38], [39], [42]. For example, *desat6* from *C. obliquana* and its ortholog from *E. postvittana (Epo-Z9*) encode a Δ9-desaturase with a preference for 18 carbon fatty acid precursors over 16 carbon precursors [39]. The remaining four desaturases all fall into the so-called Δ11 clade. While desat2 from *C. obliquana* and *C. herana* have no activity on typical sex pheromone fatty acid precursors, similar to desaturases identified previously from *Choristoneura rosaceana* and *Ch. parallela* [38], [43], we identified two desaturases showing interesting activities: desat3 and desat4. *Desat3* from *P. excessana* encodes a desaturase with terminal desaturase activity, an activity that has recently been implicated in pheromone biosynthesis in the winter moth, *Operophtera brumata* [40]. In contrast with *Ctenopseustis* and *Planotortrix*, however, the sex pheromone of the winter moth is not derived from saturated fatty acids, but from linolenic acid. *Desat4* from *C. herana* on the other hand encoded an enzyme with Δ6-desaturase activity. Like the terminal desaturase activity, this desaturation ability has only recently been observed in pheromone biosynthesis within the Lepidoptera. Wang et al. [18] described a Δ6-desaturase from the Chinese tussah silkworm, *Antheraea pernyi*, involved in the biosynthesis of the (*E*)-6-hexadecenoic acid as an intermediate in the pathway to producing the dienoic sex pheromone composed of (*E*,*Z*)-6,11-hexadecadienal, (*E*,*Z*)-6,11-hexadecadienyl acetate and (*E*,*Z*)-4,9-tetradecadienyl acetate. Despite these interesting associations with pheromone biosynthesis in other Lepidoptera, it is unlikely that desat3 and desat4, as well as desat2, are involved in pheromone biosynthesis in *Ctenopseustis* and *Planotortrix* because of their inability to produce desaturated precursors of the sex pheromone components found in the respective species, and their low level of gene expression levels in pheromone glands of all four species.

There are a number of examples of desaturase orthologs within the Lepidoptera that share a conserved biological function, especially within the two highly conserved Δ9-desaturase subfamilies (desat1 and desat6) (e.g., Roelofs et al. [19], Hao et al. [37]). Within the Δ11-like clade, in which the *desat2*, *desat3*, *desat4* and *desat5* orthologs all fall, the most widespread function is Δ11-desaturase activity. Members of this gene subfamily have been shown to evolve more rapidly than the Δ9-clades [19], which results in Δ11-like orthologs from distantly related species being less conserved (50–60%) and displaying more variable activities (i.e., Δ10, Z/E11, terminal desaturase activity, bifunctional Δ10/12 or Δ11/13). However, in closely related species the function of orthologs is usually conserved. This is the case for the Z/E11-desaturases from *Ostrinia* spp. [19], non-functional desaturases (e.g. *desat2* orthologs) from *Choristoneura* spp. [38], [43] or Z11-desaturases from *Helicoverpa* spp. [42], [44]. Only in the case of *Choristoneura parallela* and *Ch. rosaceana* did two Δ11-orthologs that shared 92% protein identity differed slightly in activity, producing E11-14:acid or a mixture of Z/E11-14:acids, respectively [38], [43]. Still both enzymes introduced double bonds at the 11^th^ carbon position, only differing in their isomeric preference. Of course one cannot rule out that some of the amino acid differences identified between the different orthologs might encode functional differences. However, we provide circumstantial evidence this is not the case from high sequence conservation (90–99% identity), from phylogenetic relationships within groups (*desat2*, *desat3*, *desat4* and *desat5*, respectively), from tests of selection and overall evidence of purifying selection on coding region sequences (Table 1, Table 2, Table 3), and from the position of the amino acid differences being outside the active site regions. We also have tested some orthologous desaturases and do not find differences in activity. Altogether, evidence from previous desaturase studies together with our sequence, phylogenetic and functional analyses provide strong support that significant functional differences between *desat3*, *desat4* and especially *desat5* orthologs are unlikely.

*Desat1*, *desat5* and *desat6* have biological activities that are sufficient to explain all but one of the observed sex pheromone components and their routes of biosynthesis (Figure 1). Foster [36] provided evidence for the role of two distinct Δ9-desaturases in the biosynthesis of the Z5-14:OAc (*desat6*) and Z7-14:OAc (*desat1*) components of the *P. excessana* sex pheromone blend and a Δ10-desaturase (*desat5*) in the biosynthesis of Z8-14:OAc in *P. octo* [35] and *C. obliquana* [33]. The exception is the biosynthesis of Z5-14:OAc in *C. herana* and *C. obliquana* that is thought to be produced directly from myristic acid by a Δ5-desaturase [34], rather than from palmitic or stearic acid via *desat1* or *desat6*, respectively, followed by rounds of chain shortening as found in *P. excessana* [35]. Despite intensive efforts, we are yet to identify another member of the fatty acyl desaturase family displaying Δ5-desaturase activity from any of the four species.

We then examined whether differential gene regulation of *desat1*, *desat5* and *desat6* could be responsible for the different sex pheromone components produced by the sibling species pairs *C. obliquana* vs *C. herana* and *P. octo* vs *P. excessana*. Interestingly, whereas *desat1* and *desat6* were expressed at largely similar levels in the four species, *desat5* showed substantial differences in gene expression in the pheromone gland of female moths between *C. obliquana* vs *C. herana*, and *P. octo* vs *P. excessana* (Figure 5). *C. obliquana* had higher levels of expression of *desat5* in female pheromone glands compared with *C. herana* and similarly, *P. octo* had higher levels of expression of *desat5* compared with *P. excessana*, which can be associated with the presence (in *C. obliquana* and *P. octo*) or absence (in *C. herana* and *P. excessana*) of the Z8-14:OAc sex pheromone component.

While we have found evidence for the role of differential gene regulation in the evolution of new pheromone blends, structural mutations have been implicated in this process in genes immediately downstream of the desaturation step within the Lepidoptera. In the European corn borer, *Ostrinia nubilalis*, a fatty-acyl reductase has been identified that alters the specificity for the desaturated precursors, leading to the production of distinct sex pheromone blends between the so-called Z and E races [45]. Since only a single reductase allele has been identified that is active in pheromone biosynthesis in these moths, with the two alleles diverging at more than 30 amino acid positions (8%) together with several coding regions being under positive selection, only structural mutations are postulated to be responsible for sex pheromone diversity at the reduction step in this species [45]. In contrast, there was no strong evidence for structural mutations being responsible for the production of different sex pheromone components by the sibling species pairs *P. octo* vs *P. excessana*, since the *desat5* genes are highly conserved (99% identity) and neither candidate loss of function mutations nor any evidence for selection acting on the coding regions of the orthologous desaturases were identified (Table 1, Table 2, Table 3). Although we miss the comparison between complete *desat5* cDNA sequences for *C. obliquana* and *C. herana*, *P. octo* and *P. excessana* differ by only two amino acid differences at the *desat5* locus. The first difference is a single amino acid deletion that occurs only in *P. octo* with respect to other *Planotortrix desat5* orthologs, and still confers this species with an intact Δ10-desaturase activity [37]. The second difference is a glutamic acid to lysine substitution two amino acids towards the C terminus from the indel. Despite the possibility that one or both of these substitutions might impact the desaturase activity of *desat5* in *P. excessana*, the fact that they lie outside active site regions suggests this is unlikely. Together, these changes in gene expression strongly suggest that a differential gene regulation of *desat5* controls the presence/absence of the Z8-14:OAc in the sex pheromone of these sibling species pairs. These results also suggest that the same molecular mechanism may have been involved independently in pheromone evolution in the two genera.

In both genera a major change in expression of *desat5* in the pheromone gland has occurred. The evolutionary event may have been either a loss of expression event from an ancestor expressing high levels of *desat5* in their pheromone glands, or a gain event with the ancestor expressing little *desat5* in their pheromone gland. We can reasonably infer the order of these evolutionary events by looking at sex pheromone composition in basal species in the two genera (see Newcomb and Gleeson [46] for discussion). Both Z8-14:OAc and Z10-16:OAc are synthesised from palmitic acid by a Δ10-desaturase (*desat5*), with Z8-14:OAc being produced following a round of β-oxidation, before reduction and acetylation (see Figure 1). Within *Ctenopseustis*, the basal species, *C. servana*, does not use any pheromone components that contain a double bond in an even position, suggesting that there has been a gain of the use of Z8-14:OAc or Z10-16:OAc early in the evolution of the genus after the split from *C. servana*. The use of these components presumably through the gain of expression of *desat5* in their pheromone glands, then become widespread, used by both *C. obliquana* (Z8-14:OAc) and *C. filicis* (Z10-16:OAc). Subsequently South Island populations of a *C. obliquana*-like ancestor lost the expression of *desat5* in the pheromone gland to give rise to *C. herana*, which only produces Z5-14:OAc. A similar loss of *desat5* expression may also have occurred in the formation of the *C. obliquana* type II that occurs in a highly restricted North Island population and also only uses Z5-14:OAc as its sex pheromone [29], [47]. The most parsimonious scenario that explains sex pheromone evolution within *Ctenopseustis* thus suggests that *desat5* pheromone gland expression was lost in *C. herana* following divergence from its *C. obliquana*-like ancestor. In contrast, the evolution of Δ10- and Δ8-unsaturated sex pheromone components within *Planotortrix* likely derives from an evolutionary scenario involving a gain of expression of *desat5* in the pheromone glands of *P. octo*. Hence in this genus, components such as Z5-14:OAc and Z7-14:OAc that are derived from the action of Δ9-desaturases are widespread within the group and thus probably represent the ancestral pheromone blend. The use of Z8-14:OAc is restricted to just two species within the genera, *P. octo* and its geographically isolated relative *P. octoides*, the latter of which is only found on the Chathman Islands. Therefore, the presence of Z8-14:OAc is a derived condition within the genus and indicates that *desat5* expression in the pheromone glands of *P. octo* is likely a gain of function event. The alternative scenario that all species but two in the *Planotortrix* genus have lost expression of *desat5* independently is a less parsimonious explanation. Simultaneously or subsequently to producing Z8-14:OAc *P. octo* must have also lost the ability to produce Z5-14:OAc and Z7-14:OAc. This may have come about through a change in one or both of the Δ9-desaturases.

Apart from this study, sex pheromone evolution through regulatory changes in desaturases has been investigated only in the corn borer moths, *Ostrinia furnacalis* and *O. scapulalis* [20]. In this example a Δ11- and Δ14-desaturase show alternate expression in the pheromone gland of the two species, with the Δ11-desaturase being expressed in the pheromone gland of *O. scapulalis* but not in *O. furnacalis* and *vice versa* for the Δ14-desaturase. Together these examples point to differential gene regulation among a standing set of desaturase genes as a mechanism involved in producing novel sex pheromone components and blends within the Lepidoptera. A further example comes from *Drosophila melanogaster* where differences in mating ability between African and cosmopolitan populations are caused by sex pheromone differences and have been suggested as a case of incipient speciation [48]. Here, a Δ9-desaturase is differentially regulated in the population through a *cis*-regulatory deletion within the promoter of the gene resulting in cuticular hydrocarbon sex pheromone differences that may ultimately promote the speciation of the two *D. melanogaster* races.

Differential gene regulation can result from two classes of mutation; either changes in the regulatory region of the differentially expressed gene (*cis*-regulatory mutation) or changes in transcriptions factors that bind to the promoter (*trans*-regulatory mutation). *Trans*-regulatory mutations are often associated with pleiotropic impacts on the regulation of other genes due to transcription factors typically acting on several promoters. Therefore it is perhaps more likely that *cis*-regulatory mutations are responsible for the differential regulation of *desat5* in *Ctenopseustis* and *Planotortrix*, an hypothesis which we are currently in the process of testing. In conclusion we show that interspecific pheromone differences between sibling species are determined by parallel changes in desaturase gene expression in two sister genera. This case study suggests that differential regulation within large multigene families may be an important process in speciation, with changes in gene expression underpinning the evolution of novel mating systems.

## Materials and Methods

### Insects

*Ctenopseustis herana*, *C. obliquana*, *Planotortrix excessana*, *P. octo* and *P. notophaea* were obtained from the Plant & Food Research insect rearing facility at the Mt Albert Research Centre, Auckland, New Zealand. The history of these strains is reported in Newcomb and Gleeson [46], except that an additional strain of *P. excessana* derived from material caught in the South Island of New Zealand was used to generate a *P. excessana* South Island strain and the original *P. excessana* strain is now known as *P. excessana* North Island. Insects were reared on a 16∶8 light cycle, with larvae reared at 20°C and pupae and adults at 18°C.

### Gene isolation

Genomic DNA was extracted using the DNeasy Blood & Tissue Kit (Qiagen). Total RNA was extracted from two distinct regions of the abdomen of 2–3 day old virgin adult females and from fat bodies of 5^th^ instar larvae. From the adult females the pheromone gland, located within the dorsal region of the 8^th^ and 9^th^ abdominal segments (denoted ‘pheromone gland’) was dissected. As a control, a lateral region of the 4^th^ to 6^th^ abdominal segments of the same adult females (denoted ‘abdomen’) was also dissected. RNA was isolated from dissected tissue using 800 µl of Trizol (Invitrogen, Carlsbad, CA, USA) following the manufacturer's instructions. The expression of desaturase genes was initially characterized from RNA generated from pools of 100 pheromone glands, while RNA from single pheromone glands was used for subsequent Quantitative Real-Time PCR experiments. After DNase treatment (DNaseI amplification grade, Invitrogen), the cDNA synthesis was carried out using the iScript cDNA Synthesis Kit (Bio-Rad) from 1 µg of total RNA or approximately 100 ng of total RNA for single samples, and incubated at 50°C for 1 hr, followed by 70°C for 15 mins.

In order to identify the different desaturases involved in sex pheromone biosynthesis, a progressive approach using successive and complementary methods was used for each species. Initially, degenerate primers were applied to genomic and pheromone gland cDNA using primers designed to conserved amino acid motifs found in lepidopteran desaturases. Sequences of the coding region of desaturase genes were then extended by means of 5′ and 3′ Rapid Amplification of cDNA ends (RACE), genome walking, or Inverse PCR. All the primers used are listed in Table S2. All PCR amplifications were performed on a GeneAmp 9700 (Applied Biosystems). The fragments of interest were cloned into pGEM-T Easy Vector System (Promega) and transformed into JM109 competent *E. coli* cells, according to the manufacturer's instructions. Sequencing was performed at the Allan Wilson Centre Genome Service (AWCGS) at Massey University, Palmerston North, New Zealand or Macrogen in South Korea.

Degenerate PCR was performed for each species on the genomic DNA or pheromone gland cDNA using 0.2 µl of Platinum *Taq* DNA polymerase (5 units/µl, Invitrogen), 1.5 mM Mg^2+^, 0.2 mM of each dNTP and 2 µM of each degenerate PCR primer (Table S2). Cycling conditions were 2 min at 94°C, 35 cycles of 94°C for 10 s, 50°C for 10 s and 72°C for 1 min, and a final extension of 72°C for 10 min.

The 3′ ends of the coding regions were obtained using a modified version of the 3′ RACE System for Rapid Amplification of cDNA ends (Invitrogen). First strand cDNA synthesis was carried out in a final volume of 14 µl using 1–2 µg total RNA, 1 µl 3′AP (or RoRidT_16_) primer (10 µM), 1 µl of 10 mM dNTPs, and incubated at 65°C for 5 min, and placed on ice for 1 min. Then, 4 µl of 5× first strand buffer, 1 µl of 0.1 M DTT and 1 µl of Superscript III (200units/µl, Invitrogen) were added to the mixture. The reactions were incubated at 50°C for 1 hr, followed by 70°C for 15 min. The 3′-tagged products were detected by PCR amplification using forward desaturase group-specific primers (3′ RACE-F primers) and the 3′AUAP (or Ri) primer, with 0.2 µl of Platinum *Taq* DNA polymerase (5units/µl, Invitrogen), 1.5 mM Mg^2+^, 0.2 mM of each dNTP and 0.2 µM of each primer. Cycling conditions were 2 min at 94°C, 30 cycles of 94°C for 10 s, 55°C for 30 s and 72°C for 1 min, and a final extension of 72°C for 10 min.

The 5′ ends of cDNAs were amplified using the 5′ RACE System for Rapid Amplification of cDNA ends kit (Invitrogen). Oligo-dC tails were added to purified 3′ RACE cDNA in a final volume of 20 µl with 4 µl of 5× first tailing buffer, 2 µl of 2 mM dCTP, 3 µl of 5 mM CoCl_2_. Reactions were incubated at 94°C for 3 min and placed on ice for 1 min. Then, 1 µl of TdT (400units/µl) was added, and the reaction was incubated at 37°C for 10 min and stopped at 65°C for 10 min. Oligo-dC-tailed products were amplified by normal PCR (30 cycles), and 1 µl of the later reaction was used for a nested-PCR (25 cycles), using the 5′ RACE-F and 5′ RACE-R primers (Table S2).

Inverse PCR was performed by digesting genomic DNA overnight at 37°C with *Nde*I or *Sal*I, BSA (100 µg/ml) and Spermidine (2 mM). Classical phenol/chloroform extraction and ethanol precipitation were used to purify the digested products. These were then circularized by ligation overnight at 16°C with T_4_ DNA ligase (400 units/µl, New England Biolabs), and purified as previously described. The 3′ ends of two distinct fragments were amplified in *C. herana* using the Inverse PCR primers (Table S2) under the same conditions as described for the 3′ RACE.

In some cases, genome walking was used to extend the coding sequences of desaturase genes. For this approach genomic DNA was digested overnight at 37°C with *Dra*I, *Eco*RV, *Pvu*II and *Stu*I, separately, and the products purified with the DNA Isolation Kit for Cells and Tissues (Roche). GenomeWalker adapters were then ligated to both ends of the digests by incubating 3 µl of template overnight at 16°C with 2 µl of adapter primers (100 µM) and 1 µl of T_4_ DNA ligase (400 units/µl, New England Biolabs) in a final volume of 20 µl. Specific tagged-products were amplified by normal and nested-PCR (see 5′ RACE), using the GW1-F and GW1-R primers (Table S2). Gene-specific PCR was conducted using highly specific PCRs (Gene-specific primers, Table S2) to verify all contigs of the coding regions.

### Bioinformatics

Sequences were analysed using Geneious Pro v5.3.4 (Biomatters). Sequences were aligned using ClustalX [49] and codon aligned nucleotide alignments were produced using RevTrans version 1.4.1 (www.cbs.dtu.dk/services/RevTrans/). For phylogenetic analyses, explicit models of evolution were determined using Modeltest [50] and GTR+I+Γ was implemented for likelihood and Bayesian analyses. Parsimony and maximum likelihood analyses were performed in PAUP v4.0b (Sinauer Associates, Sunderland, Massachusetts), and Bayesian inference was implemented in MrBayes v3.0b4 [51]. Evidence for selection was tested by looking for deviations from neutral expectations using the CODEML program in the PAML package [41]. Multiple models were run (M0, M3, M7, M8 and M8a) to assess selection pressures. Comparisons of nested models were used to assess heterogeneous selective pressure amongst sites (M0–M3) or positive selection (M7–M8). Nested models were compared by the implementation of a likelihood-ratio test (LRT), where the LRT is twice the log-likelihood difference of the nested models. Significance was tested using a χ^2^ test with degrees of freedom equal to the difference in the number of parameters between the two models.

### Quantitative real-time RT–PCR

Pheromone glands were dissected from two-day-old virgin adult females of *C. herana*, *C. obliquana*, *P. excessana* and *P. octo*. Pheromone glands were pooled in lots of 100 or used singly in RNA extractions. Fat bodies were dissected from 5^th^ instar larvae, and pooled in lots of five, while abdomens were extracted singly. RNA and cDNA were extracted as described in the gene isolation section.

The expression of the desaturases together with the housekeeper genes actin, α-tubulin and elongation factor 1 α were determined using primers described in Table S2. All quantitative real-time PCRs were performed for the pheromone gland and the abdomen, for each specimen, in duplicate. Experiments were performed on the LightCycler480 Real-Time Instrument (Roche Diagnostics, Basel, Switzerland), in a final reaction volume of 10 µL, with 80 ng of cDNA, 5 µL 2× SYBR Green Mix (Bio-Rad), and 0.5 µM of each primer. The PCR cycling conditions were set to 2 min at 95°C followed by 45 cycles of 15 s at 95°C, 30 s at 60°C and 30 s at 72°*C*. A final dissociation curve analysis was added (15 s at 95°C, 15 s at 60°C, and a gradual heating to 95°C at 0.01°C/s) to confirm the presence of a single amplicon.

Relative expression levels were calculated according to the ΔΔCp method [52], [53]. The amplification efficiency was calculated for each PCR using the LinRegPCR software [54]. For each sample, the average Threshold Cycle values were extracted, and a normalization factor, based on the geometric averaging of the reference gene expression levels, was determined using geNorm [55]. Normalization factors allowed correction for PCR efficiency and normalization of the gene expression levels. To test for differences in levels of normalised relative expression between species and tissues one way ANOVAs were conducted using GraphPad Prism 5, with individual comparisons made using Bonferroni-corrected t-tests at the 95% significance level.

### Desaturase activity assessment in yeast

Functional assays were performed with at least one ortholog of each of the desaturases. The Cob-desat2, Cher-desat2, Pex-desat3 and Cher-desat4 ORFs were cloned into pYEX-CHT vectors and transformed into the desaturase- and elongase-deficient (*elo1 ole1*) strain of *Saccharomyces cerevisiae* (*MATa elo1*::*HIS3 ole1*::*LEU2 ade2 his3 leu2 ura3*) [52]. In the same way, the Cob-desat6 ORF was cloned into the YEpOLEX vector and transformed in the desaturase-deficient (*ole1*) strain of *S. cerevisiae* (*MATα ole1Δ::LEU2 leu2-3 leu2-112 trp1-1 ura3-52 his4*) [56], [57]. Functional assays were performed with the *S.c*. Easy Transformation kit (Invitrogen AB, Lidingö, Sweden). pYEX-CHT and YEpOLEX vectors only were used as negative control.

Transformed *ole1 elo1* or *ole1* yeast cells were incubated for 4 days at 30°C on selective medium plates containing 0.7% YNB (w/o amino acids, with ammonium sulphate) and a complete drop-out medium lacking uracil and leucine (ForMedium LTD, Norwich, England), 2% glucose, 0.01% adenine, 1% tergitol (type Nonidet NP-40, Sigma-Aldrich Sweden AB, Stockholm, Sweden) and 0.5 mM unsaturated oleic acid (Larodan Fine Chemicals, Malmö, Sweden). Note that in addition to Z9-18:Acid, *ole1 elo1* yeast contained residual traces of Z9-16:Acid because of supplementation during the earlier procedure of making cells competent.

Individual *ole1 elo1* colonies were selected and incubated in 10 ml fresh selective medium and cultures were incubated in inclined position at 30°C for 48 hr and 250 rpm (Innova 42, New Brunswick Scientific). Yeast cultures were diluted to an OD_600_  =  0.4 in 10 ml fresh SC-U medium and supplemented with 25 µl CuSO_4_ 1 M in water (final concentration: 2.5 mM). After 48 hr of incubation at 250 rpm in presence of copper, yeast cells were collected by centrifugation at 2,000× g (Labofuge 200, Heraeus Instruments) and washed with sterile water. The total yeast lipid fraction was extracted with chloroform∶methanol (2∶1, v∶v) and the extracts were base-methanolyzed according to standard protocols [17], [58]. Double bond localization in methyl esters was determined by dimethyl disulfide (DMDS) derivatization [59] before GC-MS analysis.

To test for genetic complementation of the *ole1* auxotrophy by the YEpOLEX-Cob-desat6, individual SC-U-Leu yeast colonies were selected and patched onto YPAD plates lacking Fatty Acids (FA) and incubated for 4 days at 30°C. Positive transformants were subsequently grown for 48 hr at 30°C and 300 rpm in 10 ml SC medium without FA, recovered by centrifugation and washed with water, followed by base methanolysis and DMDS derivatization.

Before analysis by GC-MS analyses, samples were concentrated under a gentle flow of pure nitrogen to a final volume of approx. 50 µl. For analysis of fatty acid methyl esters (FAMEs), 3 µl was injected on a gas chromatograph (Hewlett Packard HP 5890II GC system) coupled to a mass selective detector (HP 5972) and equipped with a polar INNOWAX column (100% polyethylene glycol, 30 m×0.25 mm×0.25 µm, Agilent Technologies). The GC-MS was operated in electron impact mode (70 eV) and the injector was configured in splitless mode at 220°C with helium used as carrier gas (velocity: 30 cm/s). The oven temperature was maintained for 2 min at 50°C and increased at a rate of 10°C/min up to 220°C, held for 20 min.

For analysis of DMDS adducts, 2 µl was injected on a GC (Hewlett Packard HP 6890, Agilent Technologies) equipped with an HP-5MS capillary column (5% Phenyl Methyl Siloxane; 30 m×250 µm: df  =  0.25 µm; carrier gas: helium; velocity: 30 cm/s), an automatic injector (HP-7683), and coupled to a HP 5973 mass selective detector. The injector was configured in splitless mode at 250°C. The oven temperature was maintained for 2 min at 80°C, increased at a rate of 15°C/min up to 140°C, increased at a rate of 5°C/min up to 280°C, and held for 10 min.

## Supporting Information

Figure S1 Amino acid alignment of desat1-6 from *Ctenopseustis obliquana* (Cobl), *C. herana* (Cher), *Planotortrix octo* (Poct), *P. excessana* North Island (PexcN), *P. excessana* South Island (PexcS) and *P. notophaea* (Pnot).
(PDF)

Table S1 Sex pheromone blends used by species within the genera *Ctenopseustis* and *Planotortrix*.
(DOCX)

Table S2 Polymerase Chain Reaction primers used in this study.
(DOCX)

Text S1 Amino acid alignments of desat1 orthologs, among species within the genera *Ctenopseustis* and *Planotortrix*. Variable amino acids are in black, while invariant positions are in grey. The positions of introns are noted above the alignment with phase indicated in brackets.
(PDF)

Text S2 Amino acid alignments of desat2 orthologs, among species within the genera *Ctenopseustis* and *Planotortrix*. Variable amino acids are in black, while invariant positions are in grey. The positions of introns are noted above the alignment with phase indicated in brackets.
(PDF)

Text S3 Amino acid alignments of desat3 orthologs, among species within the genera *Ctenopseustis* and *Planotortrix*. Variable amino acids are in black, while invariant positions are in grey. The positions of introns are noted above the alignment with phase indicated in brackets.
(PDF)

Text S4 Amino acid alignments of desat4 orthologs, among species within the genera *Ctenopseustis* and *Planotortrix*. Variable amino acids are in black, while invariant positions are in grey. The positions of introns are noted above the alignment with phase indicated in brackets.
(PDF)

Text S5 Amino acid alignments of desat5 orthologs, among species within the genera *Ctenopseustis* and *Planotortrix*. Variable amino acids are in black, while invariant positions are in grey. The positions of introns are noted above the alignment with phase indicated in brackets.
(PDF)

Text S6 Amino acid alignments of desat6 orthologs, among species within the genera *Ctenopseustis* and *Planotortrix*. Variable amino acids are in black, while invariant positions are in grey. The positions of introns are noted above the alignment with phase indicated in brackets.
(PDF)
