# Supplementary material for: Sex Pheromone Evolution Is Associated with Differential Regulation of the Same Desaturase Gene in Two Genera of Leafroller Moths
Source: PLoS Genet. 2012 Jan 26;8(1):e1002489. doi: 10.1371/journal.pgen.1002489 (PMC3266893; doi:10.1371/journal.pgen.1002489)
Supplement: Figure S1 — Amino acid alignment of desat1-6 from Ctenopseustis obliquana (Cobl), C. herana (Cher), Planotortrix octo (Poct), P. excessana North Island (PexcN), P. excessana South Island (PexcS) and P. notophaea (Pnot). (PDF) [file pgen.1002489.s001.pdf]

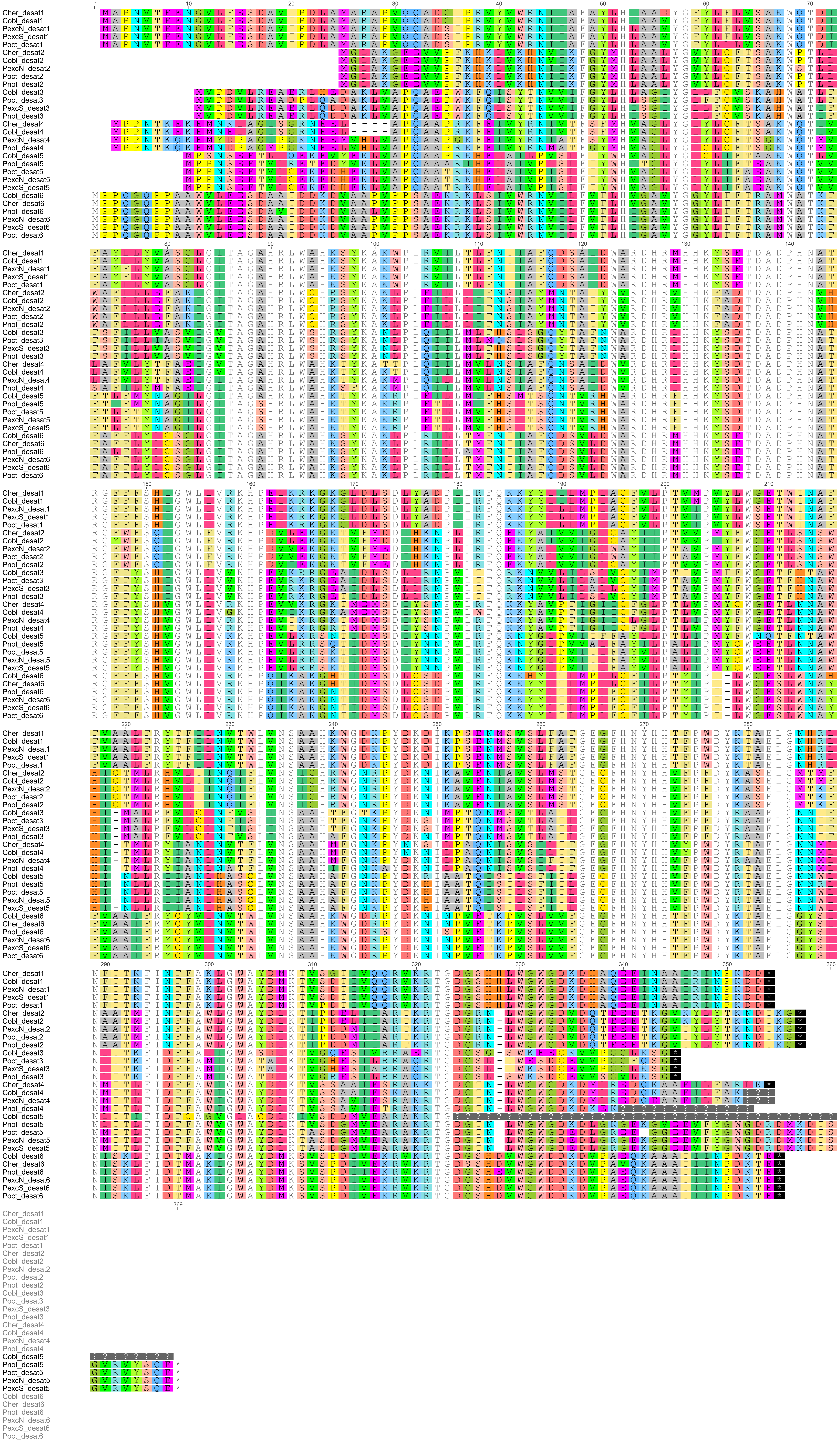

Cher_desat1
Cobl_desat1
PexcN_desat1
PexcS_desat1
Poct_desat1
Cher_desat2
Cobl_desat2
PexcN_desat2
Poct_desat2
Pnot_desat2
Cobl_desat3
Poct_desat3
PexcS_desat3
Pnot_desat3
Cher_desat4
Cobl_desat4
PexcN_desat4
Pnot_desat4
Cobl_desat5
Pnot_desat5
Poct_desat5
PexcN_desat5
PexcS_desat5
Cobl_desat6
Cher_desat6
Pnot_desat6
PexcN_desat6
PexcS_desat6
Poct_desat6
1
10
20
30
40
50
60
70
80
90
100
110
120
130
140
150
160
170
180
190
200
210
220
230
240
250
260
270
280
290
300
310
320
330
340
350
360
369
