# Supplementary material for: Sex Pheromone Evolution Is Associated with Differential Regulation of the Same Desaturase Gene in Two Genera of Leafroller Moths
Source: PLoS Genet. 2012 Jan 26;8(1):e1002489. doi: 10.1371/journal.pgen.1002489 (PMC3266893; doi:10.1371/journal.pgen.1002489)
Supplement: Table S1 — Sex pheromone blends used by species within the genera Ctenopseustis and Planotortrix. (DOCX) [file pgen.1002489.s002.docx]

**Table S1:** Sex pheromone blends used by species within the genera *Ctenopseustis* and *Planotortrix*

| Species | Sex pheromone components | Ratio | Reference |
| --- | --- | --- | --- |
| *Ctenopseustis obliquana* | Z5-14:OAc, Z8-14:OAc | 80:20 to 90:10 | Foster et al., 1986; Young et al., 1985 |
| *Ctenopseustis herana* | Z5-14:OAc | 100 | Foster & Roelofs, 1987 |
| *Ctenopseustis fraterna* | 16 carbon acetate diene ? | ? | B. Morris, unpublished preliminary data |
| *Ctenopseustis filicis* | Z10-16:OAc | 100 | Foster & Dugdale, 1988 |
| *Ctenopseustis servana* | Z5-14:OAc, Z7-14:OAc | 32:68 to 35:65 | Foster & Dugdale, 1988;  Foster et al., 1990 |
| *Planotortrix excessana* | Z5-14:OAc, Z7-14:OAc | 60:40 | Foster et al., 1989, Galbreath et al. 1985 |
| *Planotortrix octo* | Z8-14:OAc, Z10-14:OAc | 98:2 | Foster et al., 1986, Galbreath et al. 1985 |
| *Planotortrix avicennia* | Z5-14:OAc | 100 | Foster & Roelofs, 1987 |
| *Planotortrix octoides* | Z8-14:OAc | 100 | Dugdale, 1990 |
| *Planotortrix puffini* | Z5-14:OAc, Z7-14:OAc, Z9-14:OAc | 3:97:2 | Foster & Dugdale, 1988 |
| *Planotortrix flammea* | Z5-14:OAc, Z7-14:OAc | 52:48 to 61:39 | Foster et al., 1990 |
| *Planotortrix notophaea* | Z7-14:OAc, + | ? | Foster et al., 1986 |
