# Supplementary material for: Sex Pheromone Evolution Is Associated with Differential Regulation of the Same Desaturase Gene in Two Genera of Leafroller Moths
Source: PLoS Genet. 2012 Jan 26;8(1):e1002489. doi: 10.1371/journal.pgen.1002489 (PMC3266893; doi:10.1371/journal.pgen.1002489)
Supplement: Table S2 — Polymerase Chain Reaction primers used in this study. (DOCX) [file pgen.1002489.s003.docx]

**Table S2:** Polymerase Chain Reaction primers used in this study

| **Name** | **Sequence (5’-3’)** | **Application** |
| --- | --- | --- |
| desat5for | ACNGCNGGNGCNCAYMGNYTNTGG | Degen PCR |
| desat3rev | TGRTGRTARTTRTGRAANCCYTCNCC | Degen PCR |
| 3’AP | GGCCACGCGTCGACTAGTAC-dT17 | 3' RACE-R |
| 3’AUAP | GGCCACGCGTCGACTAGTAC | 3' RACE-R |
| RoRidT16 | ATCGATGGTCGACGCATGCGGATCCA  AAGCTTGAATTCGAGCTC-dT16 | 3' RACE-R |
| Ri | GGATCCAAAGCTTGAATTCGAGCTC | 3' RACE-R |
| Ro | ATCGATGGTCGACGCATGCGGATCC | 3' RACE-R |
| des3’RF1 | TCCTACAAAGCTAAGCTGCC | 3' RACE-F |
| des3’RF2 | GGCTSCACCACAAGTAYAGC | 3' RACE-F |
| CORF23'for | GAATGCACCACAAGTACTCG | 3' RACE-F |
| plg23'for | TACGGTTTCAAAAAAAGTACGCAG | 3' RACE-F |
| 5’AAP | GGCCACGCGTCGACTAGTAC-dG10 | 5' RACE-F |
| 5’AUAP | GGCCACGCGTCGACTAGTAC | 5' RACE-F |
| RF14 | AAAGAGCCATCCGATTTGCGAAAACC | 5' RACE-R |
| CHRF22 | CGTGCGAGTAGAAGAACCCCCTGG | 5' RACE-R |
| CO2rev1 | TGAACATCGTGAGCAGTATCCT | 5' RACE-R |
| RF15 | AAAGAGCCATCCGATTTGCGAGAACC | 5' RACE-R |
| PLRF2rev3 | CAGCAGCCAGCCGATGTGGGAGTAGAAG | 5' RACE-R |
| RF1irev | TTTGTAGGACCGRTGACA | Inverse PCR |
| RF1iF1 | ACGGAAGAAGAAACCAAAGGTG | Inverse PCR |
| CHiF4 | AACATTATTTATTGATTTCTTCGC | Inverse PCR,GW1-R |
| CHirev2 | CTTTGTAGGTTTTGTGAGCCCAGA | Inverse PCR,GW1-R |
| AP1 | GTAATACGACTCACTATAGGGC | GW1-F |
| AP2 | ACTATAGGGCACGCGTGGT | GW1-F |
| PERF3rev1 | ACCCTCTCCGAATGTGAAGATTGACACC | GW1-R |
| PERF3rev2 | GGTGATGTGCCAGGCGTTATTGAGCGTC | GW1-R |
| delta9for | GCGAAATGGCARACAGATAT | Gsp-desat1 |
| PromDes1R1 | CGGGGTGCTCTTCGAGAGTGACGCA | Gsp-desat1 |
| delta9rev | ATCATCTTTAGGGTTAATTCT | Gsp-desat1 |
| d11ex1F | ATGCCTCCTCAAGGGCAGCC | Gsp-desat6 |
| cD5PlaFor | ATGCCACCAAATTCAGAGGAAACAGT | Gsp-desat5 |
| cD5PlaRev | CTCTTGTGAATAAACTCTAACCCCACT | Gsp-desat5 |
| cd6rev1 | TTCAGTCTTGTCTGGGTTGATGAT | Gsp-desat6 |
| D95’probeFor | ATGGCTCCGAACGTAACTGAA | Gsp-desat6 |
| ActinFor | ACCCTAAGGCCAACAGGGAG | qPCR-HK |
| ActinRev | CAGCGAAAGTACGGCTTGGA | qPCR-HK |
| Ef1αFor | ACGTCTACAAAATCGGCGGT | qPCR-HK |
| Ef1αRev | GATGTTGGCAGGTGCGAATA | qPCR-HK |
| αTubulinFor | GACCCTCGCCACGGCAAGTA | qPCR-HK |
| αTubulinRev | GTAGGGCACCAGTCCACGAA | qPCR-HK |
| ReT9For | ACAGGCTCTGGGCACACAA | qPCR-desat1 |
| ReT9CtRev | TCTATCGCTGAGTCCTGGAATG | qPCR-desat1 |
| D5Ex3-For | GGCACTGGGCAAGGGACCAT | qPCR-desat5 |
| D5Ex3-Rev | ACCAGCAGCCAGCCTACGTG | qPCR-desat5 |
| ReTCo2For | CAAGGCCAAGGGACACACC | qPCR-desat6 |
| ReTCo2Rev | AGCACAAGAGAGGCATCAGAGTC | qPCR-desat6 |
