# Supplementary material for: Sex Pheromone Evolution Is Associated with Differential Regulation of the Same Desaturase Gene in Two Genera of Leafroller Moths
Source: PLoS Genet. 2012 Jan 26;8(1):e1002489. doi: 10.1371/journal.pgen.1002489 (PMC3266893; doi:10.1371/journal.pgen.1002489)
Supplement: Text S2 — Amino acid alignments of desat2 orthologs, among species within the genera Ctenopseustis and Planotortrix. Variable amino acids are in black, while invariant positions are in grey. The positions of introns are noted above the alignment with phase indicated in brackets. (PDF) [file pgen.1002489.s005.pdf]

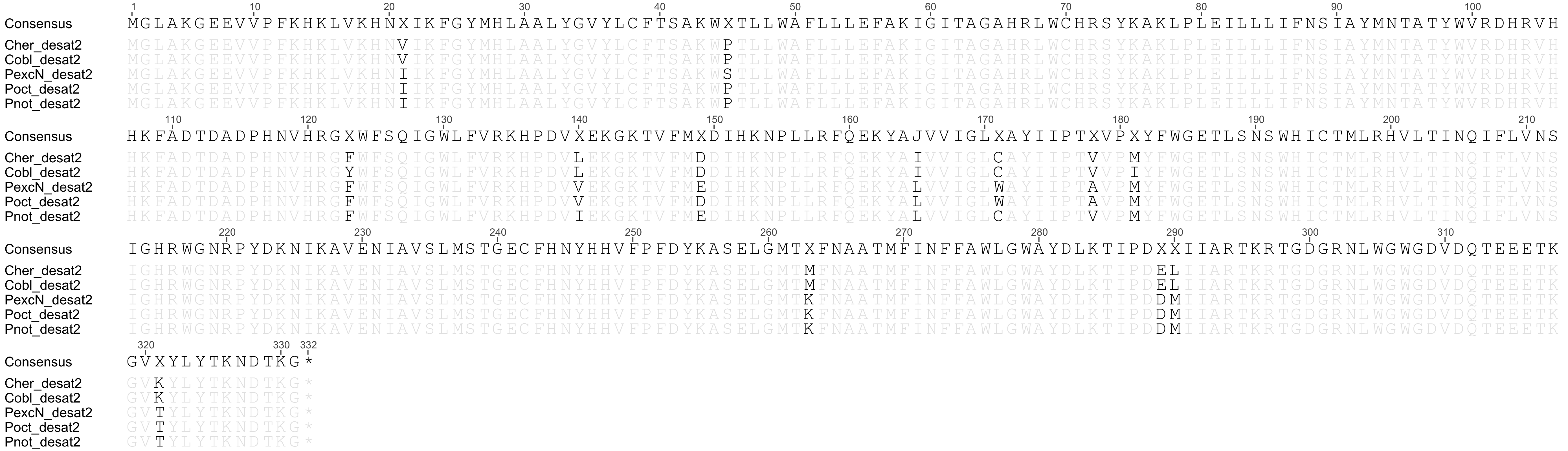
1 10 20 30 40 50 60 70 80 90 100
Consensus MGLAKGEEVVPFKHKLVKHNXIKFGYMHLAALYGVYLCFTSAKWXTLLWAFLLLEFAKIGITAGAHRLWCHRSYKAKLPLEILLLIFNSIAYMNTATYWVRDHRVH
Cher_desat2 MGLAKGEEVVPFKHKLVKHNVIKFGYMHLAALYGVYLCFTSAKWPTLLWAFLLLEFAKIGITAGAHRLWCHRSYKAKLPLEILLLIFNSIAYMNTATYWVRDHRVH
Cobl_desat2 MGLAKGEEVVPFKHKLVKHNVIKFGYMHLAALYGVYLCFTSAKWPTLLWAFLLLEFAKIGITAGAHRLWCHRSYKAKLPLEILLLIFNSIAYMNTATYWVRDHRVH
PexcN_desat2 MGLAKGEEVVPFKHKLVKHNIIKFGYMHLAALYGVYLCFTSAKWSTLLWAFLLLEFAKIGITAGAHRLWCHRSYKAKLPLEILLLIFNSIAYMNTATYWVRDHRVH
Poct_desat2 MGLAKGEEVVPFKHKLVKHNIIKFGYMHLAALYGVYLCFTSAKWPTLLWAFLLLEFAKIGITAGAHRLWCHRSYKAKLPLEILLLIFNSIAYMNTATYWVRDHRVH
Pnot_desat2 MGLAKGEEVVPFKHKLVKHNIIKFGYMHLAALYGVYLCFTSAKWPTLLWAFLLLEFAKIGITAGAHRLWCHRSYKAKLPLEILLLIFNSIAYMNTATYWVRDHRVH
110 120 130 140 150 160 170 180 190 200 210
Consensus HKFADTDADPHNVHRGXWFSQIGWLFVRKHPDVXEKGKTVFMXDIHKNPLLRFQEKYAJVVIGLXAYIIPTXVPXYFWGETLSNSWHICTMLRHVLTINQIFLVNS
Cher_desat2 HKFADTDADPHNVHRGFWFSQIGWLFVRKHPDVLEKGKTVFMDDIHKNPLLRFQEKYAIVVIGLCAYIIPTVVPMYFWGETLSNSWHICTMLRHVLTINQIFLVNS
Cobl_desat2 HKFADTDADPHNVHRGYWFSQIGWLFVRKHPDVLEKGKTVFMDDIHKNPLLRFQEKYAIVVIGLCAYIIPTVVPIYFWGETLSNSWHICTMLRHVLTINQIFLVNS
PexcN_desat2 HKFADTDADPHNVHRGFWFSQIGWLFVRKHPDVVEKGKTVFMEDIHKNPLLRFQEKYALVVIGLWAYIIPTAVPMYFWGETLSNSWHICTMLRHVLTINQIFLVNS
Poct_desat2 HKFADTDADPHNVHRGFWFSQIGWLFVRKHPDVVEKGKTVFMDDIHKNPLLRFQEKYALVVIGLWAYIIPTAVPMYFWGETLSNSWHICTMLRHVLTINQIFLVNS
Pnot_desat2 HKFADTDADPHNVHRGFWFSQIGWLFVRKHPDVIEKGKTVFMEDIHKNPLLRFQEKYALVVIGLCAYIIPTVVPMYFWGETLSNSWHICTMLRHVLTINQIFLVNS
220 230 240 250 260 270 280 290 300 310
Consensus IGHRWGNRPYDKNIKAVENIAVSLMSTGECFHNYHHVFPFDYKASELGMTXFNAATMFINFFAWLGWAYDLKTIPDXXIIARTKRTGDGRNLWGWGDVDQTEEETK
Cher_desat2 IGHRWGNRPYDKNIKAVENIAVSLMSTGECFHNYHHVFPFDYKASELGMTMFNAATMFINFFAWLGWAYDLKTIPDELIIARTKRTGDGRNLWGWGDVDQTEEETK
Cobl_desat2 IGHRWGNRPYDKNIKAVENIAVSLMSTGECFHNYHHVFPFDYKASELGMTMFNAATMFINFFAWLGWAYDLKTIPDELIIARTKRTGDGRNLWGWGDVDQTEEETK
PexcN_desat2 IGHRWGNRPYDKNIKAVENIAVSLMSTGECFHNYHHVFPFDYKASELGMTKFNAATMFINFFAWLGWAYDLKTIPDDMIIARTKRTGDGRNLWGWGDVDQTEEETK
Poct_desat2 IGHRWGNRPYDKNIKAVENIAVSLMSTGECFHNYHHVFPFDYKASELGMTKFNAATMFINFFAWLGWAYDLKTIPDDMIIARTKRTGDGRNLWGWGDVDQTEEETK
Pnot_desat2 IGHRWGNRPYDKNIKAVENIAVSLMSTGECFHNYHHVFPFDYKASELGMTKFNAATMFINFFAWLGWAYDLKTIPDDMIIARTKRTGDGRNLWGWGDVDQTEEETK
320 330 332
Consensus GVXYLYTKNDTKG*
Cher_desat2 GVKYLYTKNDTKG*
Cobl_desat2 GVKYLYTKNDTKG*
PexcN_desat2 GVTYLYTKNDTKG*
Poct_desat2 GVTYLYTKNDTKG*
Pnot_desat2 GVTYLYTKNDTKG*
