# Supplementary material for: Sex Pheromone Evolution Is Associated with Differential Regulation of the Same Desaturase Gene in Two Genera of Leafroller Moths
Source: PLoS Genet. 2012 Jan 26;8(1):e1002489. doi: 10.1371/journal.pgen.1002489 (PMC3266893; doi:10.1371/journal.pgen.1002489)
Supplement: Text S3 — Amino acid alignments of desat3 orthologs, among species within the genera Ctenopseustis and Planotortrix. Variable amino acids are in black, while invariant positions are in grey. The positions of introns are noted above the alignment with phase indicated in brackets. (PDF) [file pgen.1002489.s006.pdf]

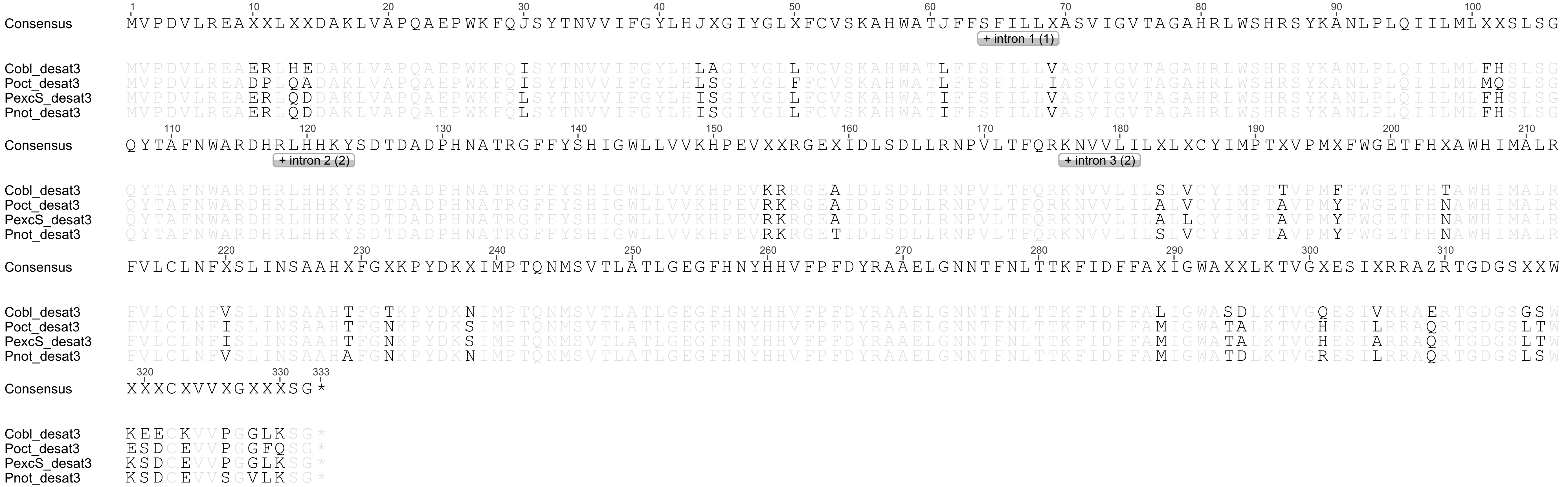

Consensus MVPDVLREAXXLXXDAKLVAPQAEPWKFQJSYTNVVIFGYLHJXGIYGLXFCVSKAHWATJFFSFILLXASVIGVTAGAHRLWSHRSYKANLPLQIILMLXXSLSG
+ intron 1 (1)
Cobl_desat3 MVPDVLREAERLHEDAKLVAPQAEPWKFQISYTNVVIFGYLHLAGIYGLLFCVSKAHWATLFFSFILLVASVIGVTAGAHRLWSHRSYKANLPLQIILMLFHSLSG
Poct_desat3 MVPDVLREADPLQADAKLVAPQAEPWKFQISYTNVVIFGYLHLSGIYGLFFCVSKAHWATLFFSFILLIASVIGVTAGAHRLWSHRSYKANLPLQIILMLMQSLSG
PexcS_desat3 MVPDVLREAERLQDDAKLVAPQAEPWKFQLSYTNVVIFGYLHISGIYGLLFCVSKAHWATIFFSFILLVASVIGVTAGAHRLWSHRSYKANLPLQIILMLFHSLSG
Pnot_desat3 MVPDVLREAERLQDDAKLVAPQAEPWKFQLSYTNVVIFGYLHISGIYGLLFCVSKAHWATIFFSFILLVASVIGVTAGAHRLWSHRSYKANLPLQIILMLFHSLSG
Consensus QYTAFNWARDHRLHHKYSDTDADPHNATRGFFYSHIGWLLVVKHPEVXXRGEXIDLSDLLRNPVLTFQRKNVVLILXLXCYIMPTXVPMXFWGETFHXAWHIMALR
+ intron 2 (2)
+ intron 3 (2)
Cobl_desat3 QYTAFNWARDHRLHHKYSDTDADPHNATRGFFYSHIGWLLVVKHPEVKRRGEAIDLSDLLRNPVLTFQRKNVVLILSLVCYIMPTTVPMFFWGETFHTAWHIMALR
Poct_desat3 QYTAFNWARDHRLHHKYSDTDADPHNATRGFFYSHIGWLLVVKHPEVRKRGEAIDLSDLLRNPVLTFQRKNVVLILALVCYIMPTAVPMYFWGETFHNAWHIMALR
PexcS_desat3 QYTAFNWARDHRLHHKYSDTDADPHNATRGFFYSHIGWLLVVKHPEVRKRGEAIDLSDLLRNPVLTFQRKNVVLILALLCYIMPTAVPMYFWGETFHNAWHIMALR
Pnot_desat3 QYTAFNWARDHRLHHKYSDTDADPHNATRGFFYSHIGWLLVVKHPEVRKRGETIDLSDLLRNPVLTFQRKNVVLILSLVCYIMPTAVPMYFWGETFHNAWHIMALR
Consensus FVLCLNFXSLINSAAHXFGXKPYDKXIMPTQNMSVTLATLGEGFHNYHHVFPFDYRAAELGNNTFNLTTKFIDFFAXIGWAXXLKTVGXESIXRRAZRTGDGSXXW
Cobl_desat3 FVLCLNFVSLINSAAHTFGTKPYDKNIMPTQNMSVTLATLGEGFHNYHHVFPFDYRAAELGNNTFNLTTKFIDFFALIGWASDLKTVGQESIVRRAERTGDGSGSW
Poct_desat3 FVLCLNFISLINSAAHTFGNKPYDKSIMPTQNMSVTLATLGEGFHNYHHVFPFDYRAAELGNNTFNLTTKFIDFFAMIGWATALKTVGHESILRRAQRTGDGSLTW
PexcS_desat3 FVLCLNFISLINSAAHTFGNKPYDKSIMPTQNMSVTLATLGEGFHNYHHVFPFDYRAAELGNNTFNLTTKFIDFFAMIGWATALKTVGHESIARRAQRTGDGSLTW
Pnot_desat3 FVLCLNFVSLINSAAHAFGNKPYDKNIMPTQNMSVTLATLGEGFHNYHHVFPFDYRAAELGNNTFNLTTKFIDFFAMIGWATDLKTVGRESILRRAQRTGDGSLSW
Consensus XXXCXVVXGXXXSG*
Cobl_desat3 KEECKVVPGGLKSG*
Poct_desat3 ESDCEVVPGGFQSG*
PexcS_desat3 KSDCEVVPGGLKSG*
Pnot_desat3 KSDCEVVSGVLKSG*
