# Supplementary material for: Sex Pheromone Evolution Is Associated with Differential Regulation of the Same Desaturase Gene in Two Genera of Leafroller Moths
Source: PLoS Genet. 2012 Jan 26;8(1):e1002489. doi: 10.1371/journal.pgen.1002489 (PMC3266893; doi:10.1371/journal.pgen.1002489)
Supplement: Text S4 — Amino acid alignments of desat4 orthologs, among species within the genera Ctenopseustis and Planotortrix. Variable amino acids are in black, while invariant positions are in grey. The positions of introns are noted above the alignment with phase indicated in brackets. (PDF) [file pgen.1002489.s007.pdf]

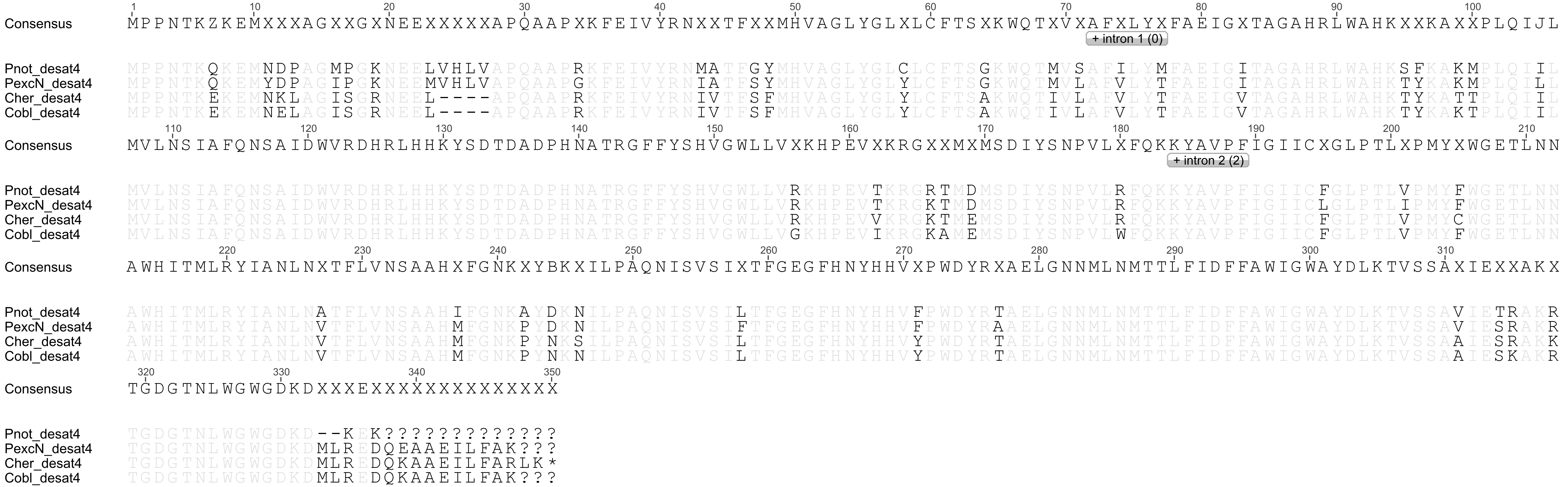
Consensus
MPPNTKZKEMXXXAGXXGXNEEXXXXXAPQAAPXKFEIVYRNXXTFXXMHVAGLYGLXLCFTSXKWQTXVXAFXLYXFAEIGXTAGAHRLWAHKXXKAXXPLQIJL
+ intron 1 (0)
MVLNSIAFQNSAIDWVRDHRLHHKYSDTDADPHNATRGFFYSHVGWLLVXKHPEVXKRGXXMXMSDIYSNPVLXFQKKYAVPFIGIICXGLPTLXPMYXWGETLNN
+ intron 2 (2)
AWHITMLRYIANLNXTFLVNSAAHXFGNKXYBKXILPAQNISVSIXTFGEGFHNYHHVXPWDYRXAELGNNMLNMTTLFIDFFAWIGWAYDLKTVSSAXIEXXAKX
TGDGTNLWGWGDKDXXXEXXXXXXXXXXXXX
Pnot_desat4
PexcN_desat4
Cher_desat4
Cobl_desat4
