# Supplementary material for: Sex Pheromone Evolution Is Associated with Differential Regulation of the Same Desaturase Gene in Two Genera of Leafroller Moths
Source: PLoS Genet. 2012 Jan 26;8(1):e1002489. doi: 10.1371/journal.pgen.1002489 (PMC3266893; doi:10.1371/journal.pgen.1002489)
Supplement: Text S5 — Amino acid alignments of desat5 orthologs, among species within the genera Ctenopseustis and Planotortrix. Variable amino acids are in black, while invariant positions are in grey. The positions of introns are noted above the alignment with phase indicated in brackets. (PDF) [file pgen.1002489.s008.pdf]

```
           1        10        20        30        40        50        60        70        80        90        100
Consensus  MPXNSEETXLXEXEXXXXKLVAPQAAXRXHELAIXPXSLFTYWHXXGLYGLXLIFXXAKWQTXVFTJFXYNAGILGITAGXHRLWAHKTYKAKRPLEXLLMXFHSXT
                                                                    + intron 1 (1)

Cobl_desat5   MPSNSEETLLQEKEVYEKLVAPQAAPRKHELAILPVSLFTYWHVAGLYGLCLIFTAAKWQTLVFTLFMYNAGILGITAGAHRLWAHKTYKAKRPLEILLMIFHSMT
Pnot_desat5   MPPNSEETVLRETEDYVKLVAPQAAARIHELAIVPLSLFTYWHITGLYGLYLIFTEAKWQTVVFTIFMYNAGILGITAGSHRLWAHKTYKAKRPLETLLMIFHSLT
Poct_desat5   MPPNSEETVLCEKEDHEKLVAPQAATRKHELAIVPISLFTYWHVAGLYGLYLIFAEAKWQTVVFTLFTYNAGILGITAGSHRLWAHKTYKAKRPLETLLMVFHSLT
PexcN_desat5  MPPNSEETVLCEKEDHEKLVAPQAATRKHELAIVPISLFTYWHVAGLYGLYLIFAEAKWQTVVFTLFTYNAGILGITAGSHRLWAHKTYKAKRPLETLLMVFHSLT
PexcS_desat5  MPPNSEETVLCEKEDHEKLVAPQAATRKHELAIVPISLFTYWHVAGLYGLYLIFAEAKWQTVVFTLFTYNAGILGITAGSHRLWAHKTYKAKRPLETLLMVFHSLT

           110       120       130       140       150       160       170       180       190       200       210
Consensus  SQNTVRHWARDHRFHHKYSDTDADPHNATRGFFYSHVGWLLVKKHPEVLXRSXTIDMSDIYNNPVLRFQKNYGLPVXXXFAYXLPXLIPMYXWXZTXNXAWHINLL
                           + intron 2 (2)                                        + intron 3 (2)

Cobl_desat5   SQNTVRHWARDHRFHHKYSDTDADPHNATRGFFYSHVGWLLVKKHPEVLKRSNTIDMSDIYNNPVLRFQKNYGLPVITFFAYLLPTLIPMYFWNQTFNTAWHINLL
Pnot_desat5   SQNTVRHWARDHRFHHKYSDTDADPHNATRGFFYSHVGWLLVKKHPEVLRRSQTIDMSDIYNNPVLRFQKNYGLPVVALFAYILPALIPMYCWEETLNNAWHINLL
Poct_desat5   SQNTVRHWARDHRFHHKYSDTDADPHNATRGFFYSHVGWLLVKKHPEVLRRSKTIDMSDIYNNPVLRFQKNYGLPVITLFAYVLPALIPMYCWEETLNNAWHINLL
PexcN_desat5  SQNTVRHWARDHRFHHKYSDTDADPHNATRGFFYSHVGWLLVKKHPEVLRRSKTIDMSDIYNNPVLRFQKNYGLPVITLFAYVLPALIPMYCWEETLNNAWHINLL
PexcS_desat5  SQNTVRHWARDHRFHHKYSDTDADPHNATRGFFYSHVGWLLVKKHPEVLRRSKTIDMSDIYNNPVLRFQKNYGLPVITLFAYVLPALIPMYCWEETLNNAWHINLL

           220       230       240       250       260       270       280       290       300       310
Consensus  RIIANLHASCLVNSAAHAFGNKPYDKXIAATQISTLSFITLGECFHNYHHVFPWDYRTAELGNNXLNXTTJFIDFXAXVGXAXDLKXXSDXMVEARAKRTGXXXXX

Cobl_desat5   RIIANLHASCLVNSAAHAFGNKPYDKRIAATQISTLSFITLGECFHNYHHVFPWDYRTAELGNNWLNLTTIFIDFCAGVGLACDLKIVSDDMVEARAKRTG?????
Pnot_desat5   RIIANLHASCLVNSAAHAFGNKPYDKHIAATQISTLSFITLGECFHNYHHVFPWDYRTAELGNNRLNLTTLFIDFFAWVGWAYDLKTVSDGMVEARAKRTGDGTNL
Poct_desat5   RIIANLHASCLVNSAAHAFGNKPYDKHIAATQISTLSFITLGECFHNYHHVFPWDYRTAELGNNWLNMTTLFIDFFAWVGWAYDLKTASDGMVEARAKRTGDGTNL
PexcN_desat5  RIIANLHASCLVNSAAHAFGNKPYDKHIAATQISTLSFITLGECFHNYHHVFPWDYRTAELGNNWLNMTTLFIDFFAWVGWAYDLKTASDGMVEARAKRTGDGTNL
PexcS_desat5  RIIANLHASCLVNSAAHAFGNKPYDKHIAATQISTLSFITLGECFHNYHHVFPWDYRTAELGNNWLNMTTLFIDFFAWVGWAYDLKTASDGMVEARAKRTGDGTNL

           320       330       340       350     358
Consensus  XXXXXXXXXXXXXXXXXXXXXXXXXXXXXXXXXXXXXXXX

Cobl_desat5   ????????????????????????????????????????
Pnot_desat5   WGWGDKDLGKGEKGVEEVFYGWGDRDMKDTSGVRVYSQE*
Poct_desat5   WGWGDEDLGR-EEGGEEVFYGWGDRDMKDTSGVRVYSQE*
PexcN_desat5  WGWGDEDLGRGEKGGEEVFYGWGDRDMKDTSGVRVYSQE*
PexcS_desat5  WGWGDEDLGRGEKGGEEVFYGWGDRDMKDTSGVRVYSQE*
```
