# Supplementary material for: Sex Pheromone Evolution Is Associated with Differential Regulation of the Same Desaturase Gene in Two Genera of Leafroller Moths
Source: PLoS Genet. 2012 Jan 26;8(1):e1002489. doi: 10.1371/journal.pgen.1002489 (PMC3266893; doi:10.1371/journal.pgen.1002489)
Supplement: Text S6 — Amino acid alignments of desat6 orthologs, among species within the genera Ctenopseustis and Planotortrix. Variable amino acids are in black, while invariant positions are in grey. The positions of introns are noted above the alignment with phase indicated in brackets. (PDF) [file pgen.1002489.s009.pdf]

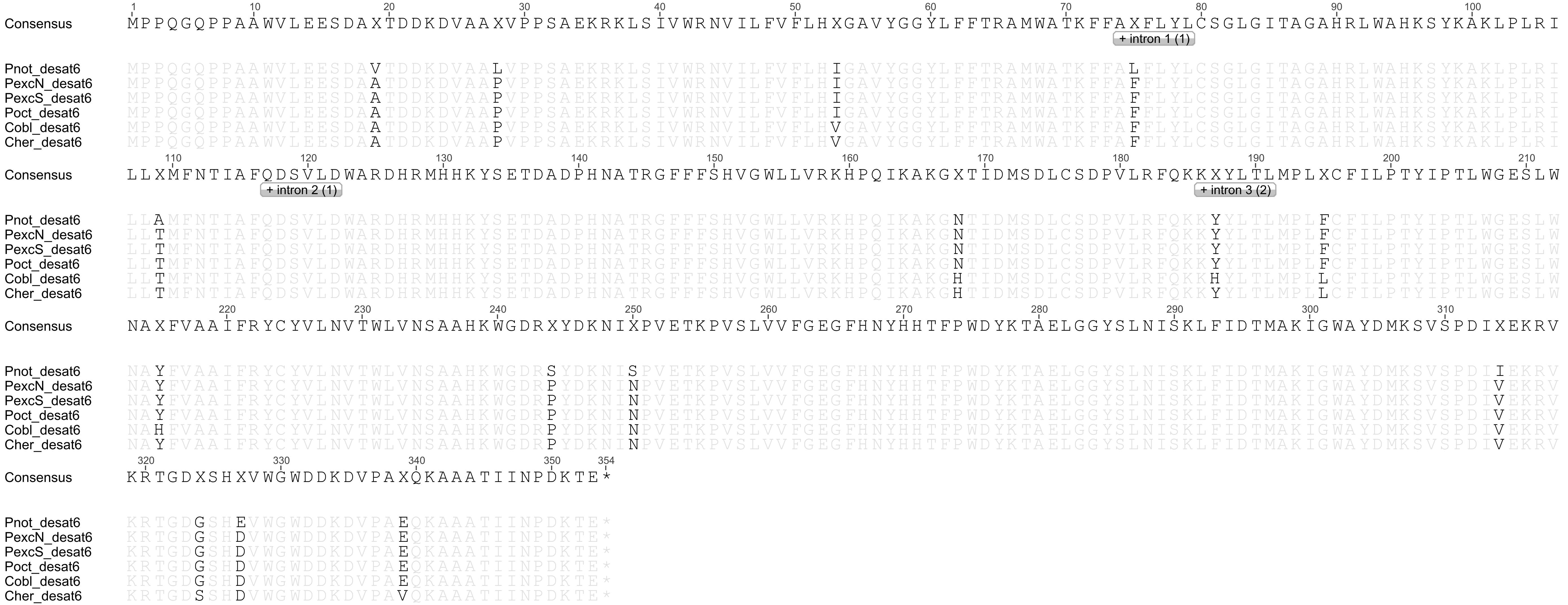
Consensus MPPQGQPPAAWVLEESDAXTDDKDVAAXVPPSAEKRKLSIVWRNVILFVFLHXGAVYGGYLFFTRAMWATKFFAXFLYLCSGLGITAGAHRLWAHKSYKAKLPLRI
+ intron 1 (1)
Pnot_desat6 MPPQGQPPAAWVLEESDAVTDDKDVAALVPPSAEKRKLSIVWRNVILFVFLHIGAVYGGYLFFTRAMWATKFFALFLYLCSGLGITAGAHRLWAHKSYKAKLPLRI
PexcN_desat6 MPPQGQPPAAWVLEESDAATDDKDVAAPVPPSAEKRKLSIVWRNVILFVFLHIGAVYGGYLFFTRAMWATKFFAFFLYLCSGLGITAGAHRLWAHKSYKAKLPLRI
PexcS_desat6 MPPQGQPPAAWVLEESDAATDDKDVAAPVPPSAEKRKLSIVWRNVILFVFLHIGAVYGGYLFFTRAMWATKFFAFFLYLCSGLGITAGAHRLWAHKSYKAKLPLRI
Poct_desat6 MPPQGQPPAAWVLEESDAATDDKDVAAPVPPSAEKRKLSIVWRNVILFVFLHIGAVYGGYLFFTRAMWATKFFAFFLYLCSGLGITAGAHRLWAHKSYKAKLPLRI
Cobl_desat6 MPPQGQPPAAWVLEESDAATDDKDVAAPVPPSAEKRKLSIVWRNVILFVFLHVGAVYGGYLFFTRAMWATKFFAFFLYLCSGLGITAGAHRLWAHKSYKAKLPLRI
Cher_desat6 MPPQGQPPAAWVLEESDAATDDKDVAAPVPPSAEKRKLSIVWRNVILFVFLHVGAVYGGYLFFTRAMWATKFFAFFLYLCSGLGITAGAHRLWAHKSYKAKLPLRI
Consensus LLXMFNTIAFQDSVLDWARDHRMHHKYSETDADPHNATRGFFFSHVGWLLVRKHPQIKAKGXTIDMSDLCSDPVLRFQKKXYLTLMPLXCFILPTYIPTLWGESLW
+ intron 2 (1)
+ intron 3 (2)
Pnot_desat6 LLAMFNTIAFQDSVLDWARDHRMHHKYSETDADPHNATRGFFFSHVGWLLVRKHPQIKAKGNTIDMSDLCSDPVLRFQKKYYLTLMPLFCFILPTYIPTLWGESLW
PexcN_desat6 LLTMFNTIAFQDSVLDWARDHRMHHKYSETDADPHNATRGFFFSHVGWLLVRKHPQIKAKGNTIDMSDLCSDPVLRFQKKYYLTLMPLFCFILPTYIPTLWGESLW
PexcS_desat6 LLTMFNTIAFQDSVLDWARDHRMHHKYSETDADPHNATRGFFFSHVGWLLVRKHPQIKAKGNTIDMSDLCSDPVLRFQKKYYLTLMPLFCFILPTYIPTLWGESLW
Poct_desat6 LLTMFNTIAFQDSVLDWARDHRMHHKYSETDADPHNATRGFFFSHVGWLLVRKHPQIKAKGNTIDMSDLCSDPVLRFQKKYYLTLMPLFCFILPTYIPTLWGESLW
Cobl_desat6 LLTMFNTIAFQDSVLDWARDHRMHHKYSETDADPHNATRGFFFSHVGWLLVRKHPQIKAKGHTIDMSDLCSDPVLRFQKKHYLTLMPLLCFILPTYIPTLWGESLW
Cher_desat6 LLTMFNTIAFQDSVLDWARDHRMHHKYSETDADPHNATRGFFFSHVGWLLVRKHPQIKAKGHTIDMSDLCSDPVLRFQKKYYLTLMPLLCFILPTYIPTLWGESLW
Consensus NAXFVAAIFRYCYVLNVTWLVNSAAHKWGDRXYDKNIXPVETKPVSLVVFGEGFHNYHHTFPWDYKTAELGGYSLNISKLFIDTMAKIGWAYDMKSVSPDIXEKRV
Pnot_desat6 NAYFVAAIFRYCYVLNVTWLVNSAAHKWGDRSYDKNISPVETKPVSLVVFGEGFHNYHHTFPWDYKTAELGGYSLNISKLFIDTMAKIGWAYDMKSVSPDIIEKRV
PexcN_desat6 NAYFVAAIFRYCYVLNVTWLVNSAAHKWGDRPYDKNINPVETKPVSLVVFGEGFHNYHHTFPWDYKTAELGGYSLNISKLFIDTMAKIGWAYDMKSVSPDIVEKRV
PexcS_desat6 NAYFVAAIFRYCYVLNVTWLVNSAAHKWGDRPYDKNINPVETKPVSLVVFGEGFHNYHHTFPWDYKTAELGGYSLNISKLFIDTMAKIGWAYDMKSVSPDIVEKRV
Poct_desat6 NAYFVAAIFRYCYVLNVTWLVNSAAHKWGDRPYDKNINPVETKPVSLVVFGEGFHNYHHTFPWDYKTAELGGYSLNISKLFIDTMAKIGWAYDMKSVSPDIVEKRV
Cobl_desat6 NAHFVAAIFRYCYVLNVTWLVNSAAHKWGDRPYDKNINPVETKPVSLVVFGEGFHNYHHTFPWDYKTAELGGYSLNISKLFIDTMAKIGWAYDMKSVSPDIVEKRV
Cher_desat6 NAYFVAAIFRYCYVLNVTWLVNSAAHKWGDRPYDKNINPVETKPVSLVVFGEGFHNYHHTFPWDYKTAELGGYSLNISKLFIDTMAKIGWAYDMKSVSPDIVEKRV
Consensus KRTGDXSHXVWGWDDKDVPAXQKAAATIINPDKTE*
Pnot_desat6 KRTGDGSHEVWGWDDKDVPAEQKAAATIINPDKTE*
PexcN_desat6 KRTGDGSHDVWGWDDKDVPAEQKAAATIINPDKTE*
PexcS_desat6 KRTGDGSHDVWGWDDKDVPAEQKAAATIINPDKTE*
Poct_desat6 KRTGDGSHDVWGWDDKDVPAEQKAAATIINPDKTE*
Cobl_desat6 KRTGDGSHDVWGWDDKDVPAEQKAAATIINPDKTE*
Cher_desat6 KRTGDSSHDVWGWDDKDVPAVQKAAATIINPDKTE*
